# NOD-like receptor repertoire in the chromosome-level genome of the demosponge *Dysidea avara* (Schmidt, 1862)

**DOI:** 10.3389/fimmu.2026.1725140

**Published:** 2026-02-03

**Authors:** Vasiliki Koutsouveli, Montserrat Torres-Oliva, Angela M. Marulanda-Gomez, Andre Franke, Janina Fuß, Ruth A. Schmitz, Ute Hentschel, Thorsten B. H. Reusch, Lucía Pita

**Affiliations:** 1 Division of Marine Ecology, Marine Evolutionary Ecology, GEOMAR Helmholtz Centre for Ocean Research Kiel, Kiel, Germany; 2 Division of Marine Ecology, Marine Symbioses Unit, GEOMAR Helmholtz Centre for Ocean Research Kiel, Kiel, Germany; 3 Institute of Clinical Molecular Biology, Kiel University, Kiel, Germany; 4 Pelagic Microbiology, Institute of Biology and Chemistry of the Marine Environment (ICBM), Carl von Ossietzky Universität Oldenburg, Oldenburg, Germany; 5 Institute of General Microbiology, Kiel University, Kiel, Germany; 6 Institute of Marine Research, Spanish National Research Council (IIM-CSIC), Integrated Marine Ecology group, Vigo, Spain

**Keywords:** chromosome-level genome assembly, comparative genomics, innate immunity, NOD-Like receptors, Porifera

## Abstract

Porifera, one of the earliest diverging metazoans, have shown a surprisingly complex immune repertoire. However, most information to date is based on *de novo* transcriptome assemblies, limiting our knowledge regarding the presence and evolution of poriferan immune repertoire. Here, we generated the chromosome-level genome of the demosponge *Dysidea avara*, a target species in studies on symbiosis and differential expression of immune genes. We examined the presence and the number of common immune protein domains in the annotated genome of *D. avara*, and we further focused on NOD-like Receptors (NLRs), which are one of the most expanded immune receptors in Porifera according to previous reports on draft genomes and transcriptome assemblies. *Dysidea avara* has a 575 Mb genome with N50 41Mb, 162 scaffolds, and 15 chromosomes. We additionally recovered 37 sequences corresponding to microbial genomes, including complete bacterial and viral genomes. Based on the presence of conserved domains, we detected a large number of immune receptors and other immune genes in *D. avara* genome, such as 14 TIR, 39 CARD, 128 DEATH, and 230 NACHT domain-containing genes. Based on their architecture, we identified a large expansion of *bona fide* NLRs (i.e., 126 NACHT+LRR domain-containing genes); of which, 20 included a N-terminal CARD domain (NLRC), and 25 included a N-terminal DEATH domain (NLRD). In *D. avara*, the different NLR categories (i.e., NLRX, NLRC and NLRD) formed distinct phylogenetic clusters, while the NLR phylogenetic analysis across sponge chromosome-level genomes indicated that NLRs were mainly grouped by species rather than category. The NLRX category was the most expanded, while the NLRC category was absent in 7 out of 11 studied sponge genomes. These observations indicate that the diversification of NLRs in sponges, most likely derived from the ancestor NLRX, responds to species-specific selective pressures related to their immunity. This is the first study characterizing sponge NLR diversity in a chromosome-level genome, enhancing our knowledge of NLR evolution in the ancient phylum Porifera.

## 1 Introduction

Porifera (sponges) is one of the earliest phyla in the evolution of metazoans (1). They are sessile organisms, dating back to 500–700 million years ago (2–4) with more than 9,300 species recorded to date (5). The phylum Porifera is divided into four different classes (Hexactinellida, Calcarea, Homoscleromorpha, and Demospongiae (6–9)), representing a crucial group for studying the evolutionary transition from unicellular to multicellular life and the emergence of metazoan traits (10). More recently, sponges have emerged as a prominent group to understand the evolution of animal-microbe interactions. Like most metazoans, sponges maintain intimate relationships with a large variety of microbes (11). It is noteworthy that sponges preserve a consistent, well-defined microbiome (12–14) while continuously filtering thousands of liters of seawater per day and encountering a wide array of foreign and potentially harmful bacteria (15). The maintenance of a stable microbiome, together with the possible recognition of distinct microbes in sponges is thought to be orchestrated by their immune system (16).

The innate immune system plays an essential role in interkingdom cross-talk and the maintenance of a healthy symbiosis between animals and microbes (17–19). Increasing evidence shows the role of host Pattern-Recognition Receptors (PRRs) in the recognition of beneficial symbionts and host-microbiome communication (16, 20). Several PRR families present recognizable architectures composed of at least one conserved protein domain that is present in homologs across the different animal groups (21). The nucleotide-binding and leucine-rich repeat receptors (NLRs) are among the best characterized family of PRRs (22). Interestingly, NLRs are especially diversified in early diverging metazoans, such as cnidarians and sponges (23–26) and in other marine invertebrates (27), and are hypothesized to play a role in invertebrate immune specificity (28, 29). NLRs are recognized by the conserved NACHT protein domain (30, 31), combined with variable C-terminal LRR domains (30). This architecture defines *bona fide* NLRs. Their canonical structure, as described in vertebrates, is complemented by an N-terminal with CARD, Death, Pyrin, or BIR domain (30, 31). NLRs in vertebrates recognize microbes via the C-terminal LRR, which bind microbial patterns (e.g., lipopolysaccharide (LPS), peptidoglycan (PGN), peptides) (32, 33). Then, the N-terminal domain participates in protein-protein interactions and is responsible for activating further downstream pathways related to inflammation during pathogen infections (34). NLRs in invertebrates seem to have a similar role, being activated in defense mechanisms (23, 35). Besides, NLRs have also been considered to play a role in differentiating mutualists from pathogens and in symbiosis establishment, for example in the intestinal microbiota in humans (36). Similarly, the expanded NLR repertoire in the coral *Acropora digitifera* have also been proposed to be devoted to interaction with their obligate dinoflagellate endosymbiont (24).

Previous studies based on draft genomes and transcriptomes have indicated a complex innate immune repertoire in sponges (11, 26, 28, 37–41). Sponges possess a relatively large expansion of *bona fide* NLR receptors, which might indicate a high level of specificity to the large range of microbes they encounter (28). Functional validation of NLR roles in sponges is limited, but bulk transcriptomics in the sponge *Dysidea avara* revealed the differential expression of NLR genes in response to a “cocktail” of microbial-associated molecular patterns (MAMPs) (26) and when incubated with sponge-associated *vs* food bacteria (42). Building on this previous knowledge, our focus here was the species *D. avara*. *Dysidea avara* is an encrusting sponge of the class Desmospongiae from the Atlantic-Mediterranean region, found in rocky sublittoral habitats, at a maximum of 80m depth (43, 44). It is characterized as Low Microbial Abundant (LMA) sponge because it harbors a low quantity of symbiotic bacteria, compared to other species (45). This species has received considerable attention due to its potential pharmaceutical value. *Dysidea avara* produces avarol and derivatives of broad-spectrum activity, including antitumor, antipsoriatic, and antileukemic properties (46, 47). Moreover, the physiology of *D. avara* and its role in benthic-pelagic nutrient transfer has been extensively studied, connecting the metabolism of the sponge and its microbiota with ecosystem processes (48–50). This species is one of the few sponge species in which larva settlement, juvenile development and metamorphosis have been achieved *in vitro* (51). Due to all the above, *D. avara* emerges as a model species for studying sponge-microbe interactions and the evolution of the innate immune system (26, 42, 52). Here, we generated a chromosome-level assembly from this species with PacBio and Hi-C technologies with the aim of identifying the presence/absence and expansion of most common metazoan immune related domains and further exploring the NLR receptor expansion and structure in this species. We further conducted a phylogenetic analysis to understand the NLR evolution and expansion within *D. avara* and in relation with other sympatric sponge species. In comparison to previous investigations based on *de novo* transcriptome assemblies (e.g. 26, 42), the use of such a chromosome-level genome assembly in NLR investigation is expected to give accurate information of the full NLR composition (and not only the expressed fraction) and the exact position of those NLR genes on the chromosomes of *D. avara.* This information can be further used to investigate evolutionary traces of the different NLR families in a broader context and among sponges but also across other animal phyla.

## 2 Materials and methods

### 2.1 Specimen collection

**Figure 1:**
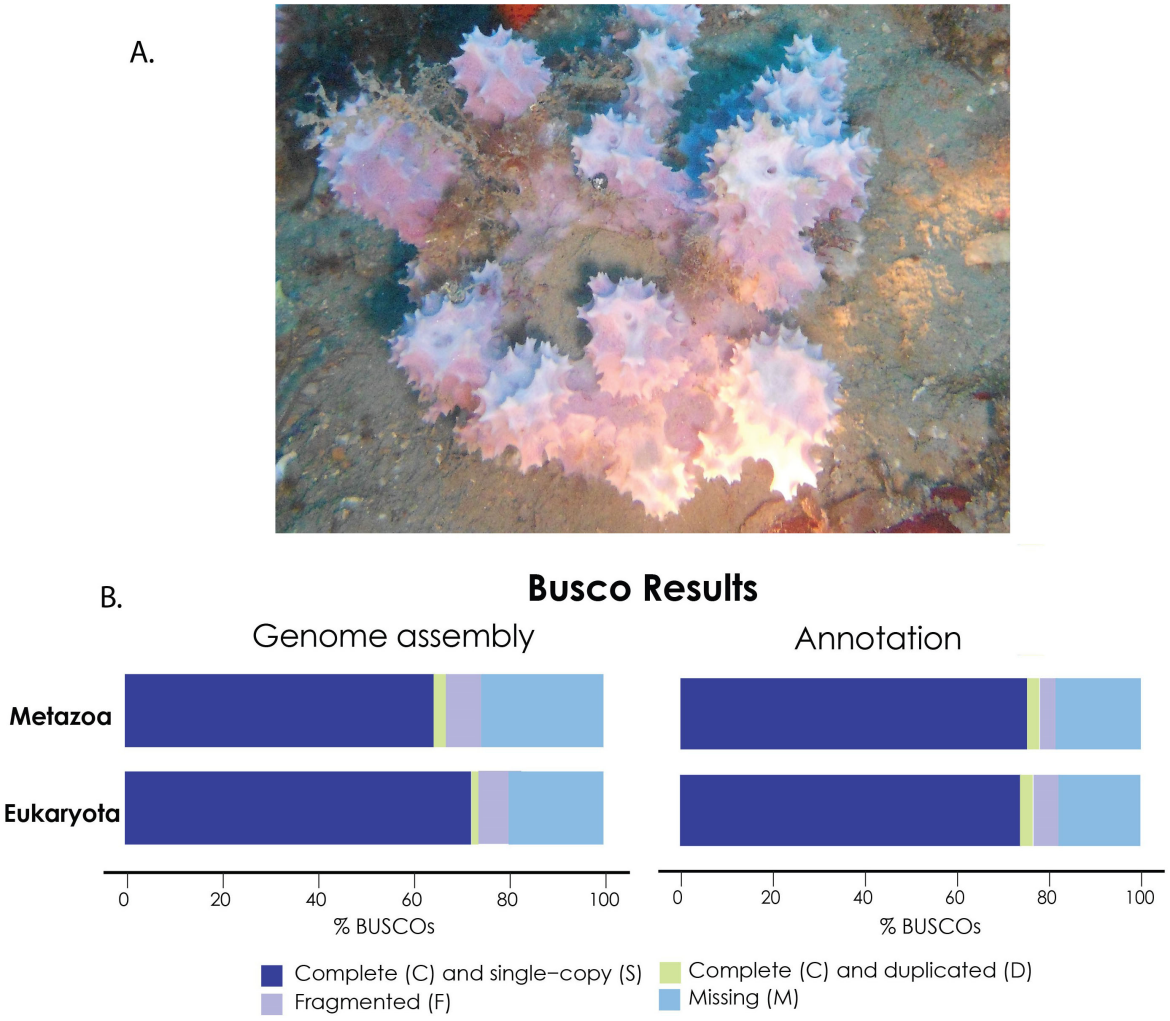
*Dysidea avara* species and its genome statistics. **(A)** Underwater photograph of the sponge *D avara*. **(B)** BUSCO scores indicating the quality of the genome and its annotation, taking into account the eukaryota_odb10 and metazoa_odb10 databases. The BUSCO analysis was run with Busco v.5 version.

An individual of *D. avara* (**Figure 1A**) was originally collected in April 2019 at l’Escala, Girona, Spain (42.1145863 N, 3.168486 E) and kept in a flow-through system with direct intake of seawater in aquaria facilities at ICM-CSIC (Barcelona, Spain) for about 1 month. Tissue sample from the sponge was then collected, cleaned from any epiphytes and snap-frozen in liquid nitrogen, followed by storage at -80 °C until further processing. We performed a sponge cell separation by differential centrifugation prior to DNA purification, adapting the protocol by Wehrl et al. (53). This step was meant to enrich the sponge cell fraction of the sample, reducing potential bacterial contamination in DNA extracts. In short, ca. 15 mL of tissue volume was cut into small pieces and incubated in calcium-magnesium-free artificial seawater with EDTA (CMFASW-E) to favor cell dissociation and gentle homogenization, and then filtered through 100 µm Nitex. The resulting suspension was centrifuged at 400x g for 10 min at 4 °C to recover the pellet with the sponge cells. The supernatant was discarded, and the pellet was re-suspended in CMFASW-E and centrifuged again to remove any remnant debris. The resulting pellet was immediately processed for DNA extraction.

### 2.2 Genome assembly and annotation

#### 2.2.1 High-molecular-weight DNA purification

High-molecular-weight DNA was extracted with the NucleoBond^®^ HMW DNA kit (Macherey-Nagel Gmbh & Co, Germany) following the manufacturer’s instructions, by adding an extra wash step with solution H4. The quality, quantity, and size of DNA extracts were assessed by NanoDrop 2000c Spectrophotometer (peolab, Germany), Qubit 2.0 (Life Technologies, Carlsband, CA), and by pulse-field gel electrophoresis in 0.75% agarose gel in 1x Lönning buffer with SYBRSafe DNA stain (1:100000; Sigma-Aldrich).

#### 2.2.2 Library construction and draft genome sequencing

Long read sequencing was performed using the PacBio Sequel II platform at CCGA sequencing facility in Kiel. Three consecutive SMRT Cells were run, obtaining a total of 32.1 Gb of CCS reads. The IPA v1.8 (https://github.com/PacificBiosciences/pbipa) assembler was run with *local* mode (including polishing, purging haplotigs, and phasing) to assemble together the reads from all the three libraries and to generate the *D. avara* draft assembly. This assembly was used as input for Hi-C sequencing as described below.

#### 2.2.3 Dovetail Omni-C library preparation and sequencing

Frozen material from the same *D. avara* individual was sent to Cantata company (previous Dovetail Genomics) for generating Omni-C^®^ libraries and scaffolding the draft genome with the HiRise™ software. For each Dovetail Omni-C library, chromatin was fixed in place with formaldehyde in the nucleus. Fixed chromatin was digested with DNase I and then extracted. Chromatin ends were repaired and ligated to a biotinylated bridge adapter followed by proximity ligation of adapter adapter-containing ends. After proximity ligation, crosslinks were reversed and the DNA purified. Purified DNA was treated to remove biotin that was not internal to ligated fragments. Sequencing libraries were generated using NEBNext Ultra enzymes and Illumina-compatible adapters. Biotin-containing fragments were isolated using streptavidin beads before PCR enrichment of each library. The library was sequenced on an Illumina HiSeqX platform to produce ~ 30x sequence coverage.

#### 2.2.4 Scaffolding with Omni-C HiRise

The input *de novo* assembly and Dovetail OmniC library reads were used as input data for HiRise, a software pipeline designed specifically for using proximity ligation data to scaffold genome assemblies (54). Dovetail OmniC library sequences were aligned to the draft input assembly using bwa (https://github.com/lh3/bwa). The separations of Dovetail OmniC read pairs mapped within draft scaffolds were analyzed by HiRise to produce a likelihood model for genomic distance between read pairs, and the model was used to identify and break putative misjoins, to score prospective joins, and make joins above a threshold. Genome completeness was assessed with BUSCO v5.3.0 (55), using both the eukaryota_odb10 and the metazoa_odb10 databases to interrogate the assembly for the presence of common single-copy orthologs. A prediction of genome size for *D. avara* was obtained using GenomeScope software (v2.0) (56) and all generated CCS reads.

#### 2.2.5 Genome annotation

The annotation of the generated chromosome-level genome of *Dysidea avara* was conducted with the *genomeannotator* pipeline developed in-house (https://github.com/marchoeppner/genomeannotator). It is an automatic genome annotation pipeline based on ab-initio gene prediction as well as experimental and additional model hints from multiple possible sources, using the Nextflow workflow language (57). We also used a subset of RNA seq data from a previous publication (26), (Experiment ArrayExpress accession E-MTAB-6757; ENA run accessions: ERR2560048; ERR2560049; ERR2560053; ERR2560055; ERR2560056).

### 2.3 Immune Pfam domain identification

#### 2.3.1 Search of immune Pfam domains in annotated *D. avara* genome

Specific domains of the metazoan immune repertoire were selected, based on the review by Buckley et al. (21), and searched in the translated genome of *D. avara*. We used the respective PFAM database entry from the Interproscan page (https://www.ebi.ac.uk/interpro/entry/pfam/) for each domain. The search in the predicted gene models was conducted with hmmerscan v.3.4 (http://hmmer.org/) with default parameters. We further checked those sequences in SMART (http://smart.embl-heidelberg.de/) keeping only the sequences above threshold that included the target Pfam domain as visible or overlapping with other domain(s) within the sequence, as determined by SMART. We expanded our analysis to 10 other translated chromosome-level sponge genomes, recently generated during Aquatic Symbiotic Project by the Wellcome Sanger Institute (ASG), (**Supplementary Table 1**). We included 4 demosponges and 1 Homoscleromorpha that can all occur in sympatry with *D. avara* in the Mediterranean Sea. We also included 2 additional demosponges that have been target species of studies on immunity (*Halichondria panicea* and *Amphimedon queenslandica*), plus 1 freshwater demosponge (*Ephydatia muelleri*) and 1 Hexactinellida (*Aphrocallistes vastus*). The ASG recently generated a chromosome-level genome of our target species, *D. avara*, which was also included in our analysis for comparison. Finally, we reported the number of genes containing the target Pfam domains for each species.

#### 2.3.2 Search and characterization of *D. avara* annotated genes coding for NLRs

We extracted the protein sequences of those genes that had similarity with NLRs from the automated eggNOG annotation of our genome. Additionally, we used the NACHT containing proteins retrieved from the previous hmmer search (section 2.3.1) in order to include more divergent *D. avara* NLRs. We checked manually for their domain architecture in SMART in Genomic mode (http://smart.embl-heidelberg.de/) (58). To illustrate the domain architecture of the candidate NLR proteins, we used the Biorender.com online software. We also checked the positions of those genes on the chromosomes of the *D. avara* genome, using the RIdeogram v0.0.2 in R (59).

#### 2.3.3 Phylogeny of poriferan NLRs

For determining the phylogeny of *D. avara* NLR genes in the context of NLR repertoires in other sponge species, we focused on their most conserved domain: the NACHT domain (30, 31). For this analysis we did a different approach in order to extract comparable information for all studied species. We first constructed a custom hmmer profile from the alignment of NACHT domains retrieved in a total of 28 sequences from different phyla of metazoans, placozoans, and other sponges from NCBI (**Supplementary Table 2A****;****Supplementary File 1**). We performed alignments with MUSCLE algorithm (60) in Geneious software (61), and selected the part of the alignment that was better aligned among all phyla (**Supplementary File 2A**). We chose MUSCLE because of its high accuracy on amino acid datasets, without incurring substantial computational cost. Checking in SMART, we further confirmed that this selected alignment was assigned to a NACHT domain. The hmmer profile was used to retrieve NACHT-containing proteins from the translated genome of *D. avara* (**Supplementary Table 2B****;****Supplementary File 1**) and the 10 other sponge translated chromosome-level genomes (**Supplementary Table 1****;****Supplementary Table 2C****;****Supplementary File 1**). The domain architectures of the retrieved NACHT-containing proteins were further checked in SMART, and only those corresponding to *bona fide* architectures (that is those combined with leucine-rich repeats) were selected for further analysis (**Supplementary File 1**). In order to generate a phylogenetic tree of *bona fide* NLRs in *D. avara*, we combined the results obtained from the hmmer search and the direct search on automated annotation (see section 3.2.3) (**Supplementary File 1****;****Supplementary File 2B**). For the NLR phylogeny across sponge species, we also included as outgroup the NLR sequences of the cnidarian species *Nematostella vectensis* (**Supplementary Table 2A****;****Supplementary File 1****;****Supplementary File 2C**). Both phylogenetic trees were constructed based on the NACHT domain alignment from all the NLR sequences either within *D. avara* or across all sponge species (**Supplementary File 1****;****Supplementary Files 2B, 2C**). The phylogenetic trees were generated by maximum likelihood in RAxML v.8 (62) with the raxmlGUI platform v2.0.1 (63), using GTR Bootstrap model for proteins and an estimated gamma shape parameter. The node support was calculated with a thorough bootstrap algorithm and 100 independent searches. The tree was further processed with figtree v.1.4.4. and the online tool iTOL (https://itol.embl.de/).

## 3 Results

### 3.1 Draft and chromosome-level genome assembly

A single *D. avara* individual from Girona, Spain (42.1145863 N, 3.168486 E) was used to generate a draft genome assembly. After extracting high molecular weight DNA, three PacBio Sequel II SMRT Cells (PacBio, Menlo Park, CA) were sequenced, generating a total of 32.1 Gb of data output in the form of CCS/HiFi reads (representing a coverage of ~55.8x based on the final assembly size). CCS/HiFi reads were assembled using the IPA pipeline, generating a draft assembly with a size of 648 Mb and a scaffold N50 of 3 Mb. The BUSCO score showed a completeness of metazoan single-copy orthologs of 86.8% and a duplication rate of 2.6%. By mapping the Omni-c libraries, generated by Cantana company, to the draft genome assembly, a high-quality chromosome-level genome assembly was generated for *D. avara* with a size 575 Mb, 162 scaffolds, and a scaffold N50 of 41Mb (**Supplementary Table 3**) structured in 15 chromosomes (**Supplementary Figure 1**). This assembly size is close to the k-mer based size prediction (499 Mb, **Supplementary Figure 2**). From the 147 scaffold sequences that were not assigned to a chromosome, 61 have a high similarity blast hit (E< 1e-30) to one of the assembled chromosomes, indicating they are alternative haplotype regions. From the remaining 86 sequences, after mapping against NCBI nr database using blast search, 37 sequences corresponded to microbial genomes, recovering the complete genomes of several bacteria, mostly of the genus Endozoicomonas (four different strains), and virus (**Supplementary Table 4**). The downstream analyses were carried out only with the sequences assigned to chromosome 1 to 15. The genome assembly was 64.9% complete according to the BUSCO score for metazoans (**Figure 1B**; **Supplementary Table 3**).

The automated genomic annotation of this chromosome-level assembly identified 31,387 protein coding genes. We were able to assign KEGG pathways to 7,284 of these gene models, GO terms to 9,514 models and PFAM domains to 18,198 gene models (**Supplementary Table 5**). BUSCO results showed that our genome annotation strategy was able to retrieve up to 77.2% of single-copy metazoan orthologs (**Figure 1B****;****Supplementary Table 3**).

Comparing the Busco statistics of our genome with the ASG generated genome assembly of *D. avara*, we found that duplication rates of the genome assemblies were 2.3% and 2.0% for our genome and the ASG genome respectively (with completeness reaching 64.9% and 74.6% respectively) (**Supplementary Table 3**). When comparing the Busco results of the annotated genomes, the duplication level was 2.3% for our annotated genome while it was 44.3% for the ASG annotated genome (**Supplementary Table 3**).

### 3.2 Repertoire of protein domains related to animal immune receptors

We examined the presence of 24 conserved Pfam domains that are related to immune receptors in metazoans (21) and reported the number of genes that contain each domain (**Table 1**). We detected all the target domains in *D. avara* genome, except for the Leucine-rich repeat C-terminal domain, LRCNT (**Table 1**). The I-set (s) and V-set Pfam domains were represented by a relatively much higher number of gene sequences (637 sequences and 594 sequences, respectively) than other Pfam domains, such as the BIR, SEFIR, LRRNT and CTLD that were present in much less sequences (15 sequences, 1 sequence, 2 sequences, and 3 sequences, respectively). The C-set and NACHT domains were also represented by a relatively high number of sequences (371 and 230 sequences, respectively) (**Table 1**). Similar patterns were observed in most other sponge species of our study (**Supplementary Table 6**). All the sequences comprising LRR_2 and LRR_3 domains were already recovered in the LRR_1 domain search, while most of the LRR domains found in *D. avara* annotated genome corresponded to LRR_4 and LRR_6 domains, according to SMART search. We also found 14 gene sequences that contained TIR Pfam domain: 6 out of the 14 TIR domain-containing genes were combined with an immunoglobulin domain and one combined the TIR domain with a DEATH domain. Finally, 19 gene sequences contained TIR2 Pfam domain (**Table 1**), and 11 of those sequences were commonly assigned to both TIR and TIR2 domains.

**Table 1:** Abundance of Pfam domains related to immunity in the genome of *Dysidea avara*.

PFAM-ID	Domain	*D. avara*
PF00001	7tm_1	8
PF00002	7tm_2	56
PF00003	7tm_3	25
PF00059	Lectin_C	3
PF00147	Fibrin C	59
PF00270	DEAD	111
PF00530	SRCR	108
PF00560	LRR_1	250
PF00619	CARD	39
PF00653	BIR	15
PF01335	DED	7
PF01462	LRRNT	1
PF01463	LRCNT	0
PF01582	TIR	14
PF13676	TIR_2	18
PF05790	C2-set	209
PF07654	C1-set	162
PF05729	NACHT	230
PF07679	I-set	637
PF07686	V-set	564
PF07723	LRR_2	9
PF07725	LRR_3	6
PF08357	SEFIR	2

The number of proteins assigned to a specific domain was taken after independent hmmerscan for each domain based on default parameters and validation with SMART. TIR, Toll/interleukine-2 domain; I-set, Immunoglobilin I-set; V-set, Immunoglobilin V-set; C1-set, Immunoglobilin C1-set, C2-set, Immunoglobilin C2-set; LRR1, Leucine-rich repeat 1 domain; LRR2, Leucin-rich repeat 2 domain; LRR3, Leucin-rich repeat 3 domain; LRRNT, Leucin-rich repeat N-terminal domain; LRCNT, Leucin-rich repeat C-terminal domain; SRCR, Scavenger Receptor cystein rich domain; CARD, Caspase recruitment domain; DEAD/DEATH box helicase; DED, Death effector domain; LCTD, Lectin C type domain; Fibrin C, Fibrinogen beta and Gamma chains C-terminal globular domain; BIR, Inhibitor of Apoptosis domain; TM, 7 transmembrane (TM) helices_1,2,3.

### 3.3 Automated characterization of *D. avara* NOD-like receptors

**Figure 2:**
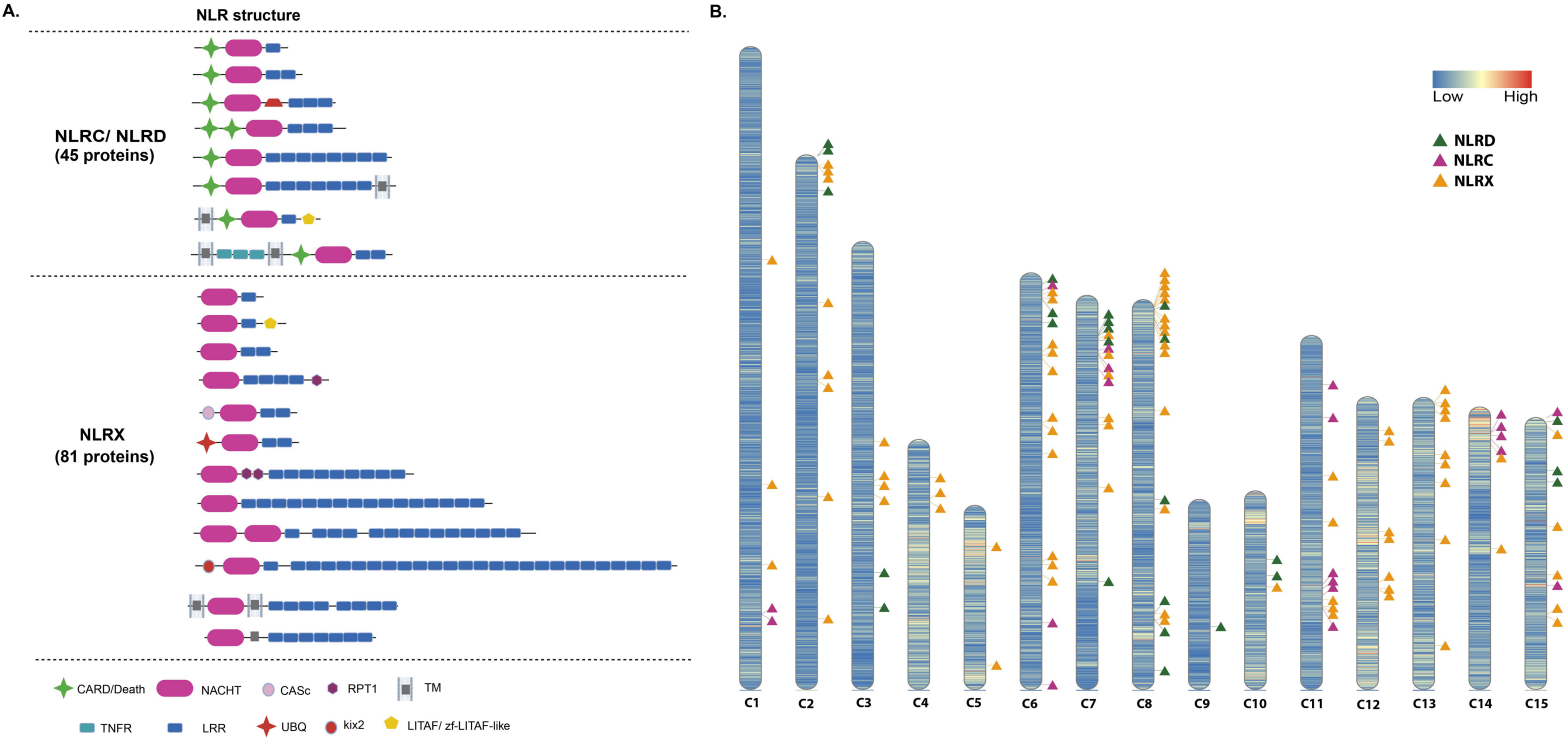
NOD-Like Receptors (NLRs) in *Dysidea avara* genome. **(A)** Domain architecture of proteins annotated to the different categories of *bona fide* NLRs (NLRD, NLRC, NLRX) in the genome assembly of *D avara*. The number indicates the annotated genes assigned to each NLR category. The graph was generated in Biorender.com. **(B)** Genes coding for *bona fide* NLRs were depicted in the chromosomes they are located in the genome. The graph was created with RIdeogram. NLRC, CARD-NACHT-LRR; NLRD, DEATH-NACHT-LRRs; and NLRX, NACH-LRR. CARD, Caspase recruitment domain; LRR, Leucine-rich repeat domain; Kix, kinase-inducible domain (KID) interacting domain; TNFR, tumor necrosis factor receptor; RPT, internal repeat; CASc, caspase; UBQ, ubiquitin; Litaf, LPS-induced TNF-activating factor; COR, C-terminal of Ras of Complex domain.

The eggNOG automated annotation of *D. avara* genome identified 242 genes with sequence similarity to NLRs. Based on the domain architecture, 126 out of these 242 genes belonged to *bona fide* NLR genes (**Supplementary Table 7**). In particular, we identified 20 NLRC, 25 NLRD, and 81 NLRX genes (**Figure 2A****;****Supplementary Table 7**). NLRX includes mainly NACHT-LRR architectures without an identified N-terminal domain, but also a few cases of N-terminal domain different from CARD and DEATH (**Figure 2A****;****Supplementary Table 7**). Those 126 genes were spread homogeneously across the 15 chromosomes of *D. avara* genome, though some NLRX genes were found forming clusters in the chromosomes (e.g. in chromosome C8; **Figure 2B****;****Supplementary Table 8**). Finally, we identified another 63 genes that had only a NACHT annotation and another 64 NACHT containing genes with domain architectures not associated to *bona fide* NLRs (i.e. NACHT-WD40; NACHT-TPR; sushi-NACHT) (**Supplementary Table 9**); thus, we did not include those in our further analysis.

### 3.4 NLR categories in *D. avara*

**Figure 3:**
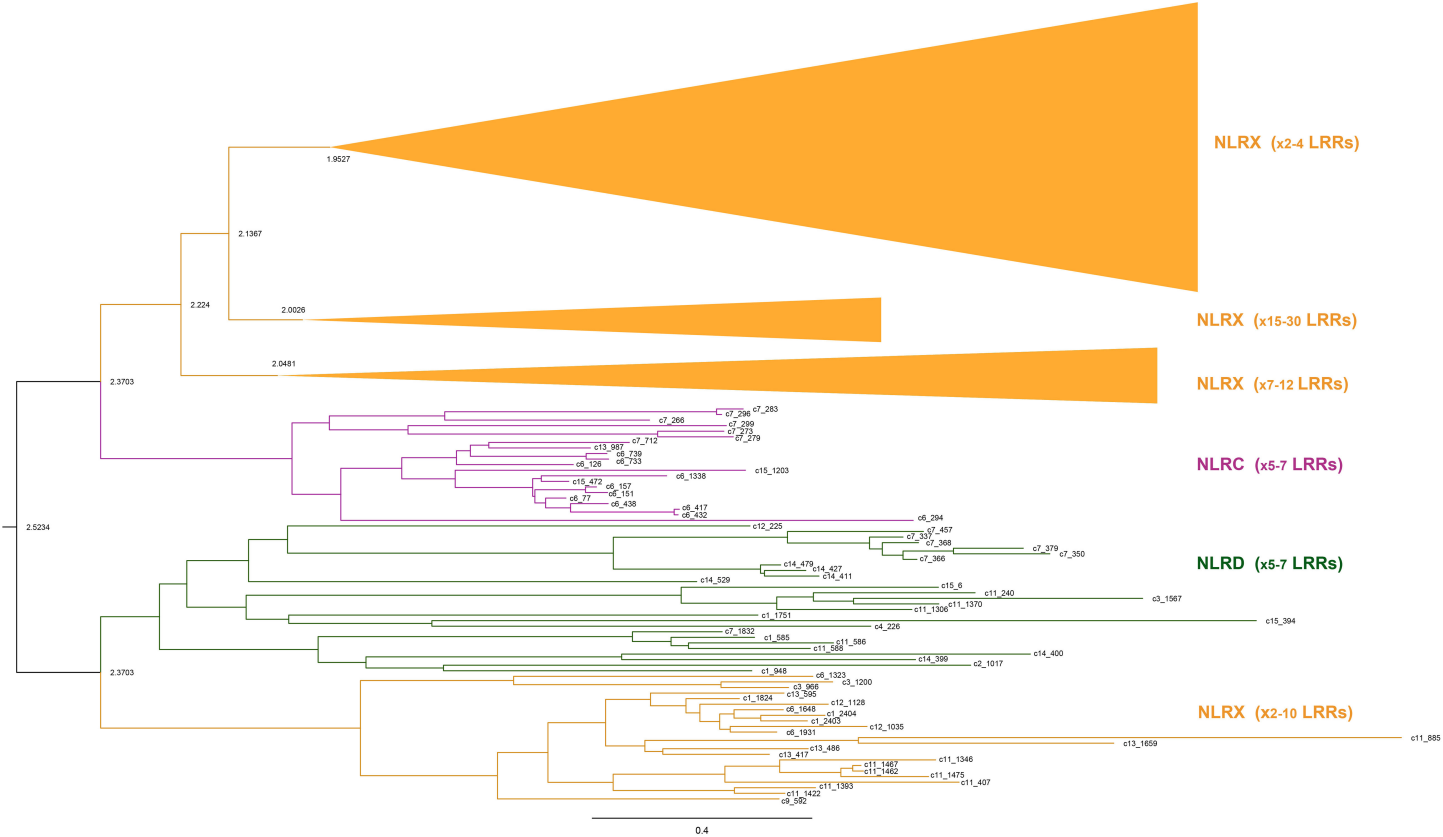
Phylogenetic analysis of NLRs in *Dysidea avara*. The NACHT domain alignment from NLR sequences was used for the analysis (**Supplementary File 1B**; **Supplementary File 2**). Different clusters were shaped, depending on the NLR category NLRD or NLRC, while NLRX was grouped with the two other groups. Within the NLRX category, the number of C-terminal LRR repeats also determined different clusters. The ML tree was constructed with GTR Bootstrap expectation model and an estimated gamma shape parameter and 100 independent searches with RAxML. NLR, NOD-Like Receptor; LRR, Leucine Rich Repeat; NLRC, CARD-NACHT-LRR; NLRD, DEATH-NACHT-LRR; and NLRX, NACHT-LRR.

We first focused on the NACHT containing proteins which make part of the different NLR categories in *D. avara*. We found that *D. avara bona fide* NLRs form two distinct large clusters separating the NLRC and NLRD categories (**Figure 3**). The NLRX category was present in both groups, but the majority of NLRX were more closely related to the NLRC category. Interestingly, those NLRX closer related to NLRC were clustered according to the number of C-terminal LRR repeats: a group of NLRX with low number of LRR repeats (2–4 repeats), another cluster with NLRX with 7–22 LRR repeats and finally a cluster with 15–30 LRR repeats (**Figure 3**).

### 3.5 Phylogeny of poriferan *bona fide* NLRs

**Figure 4:**
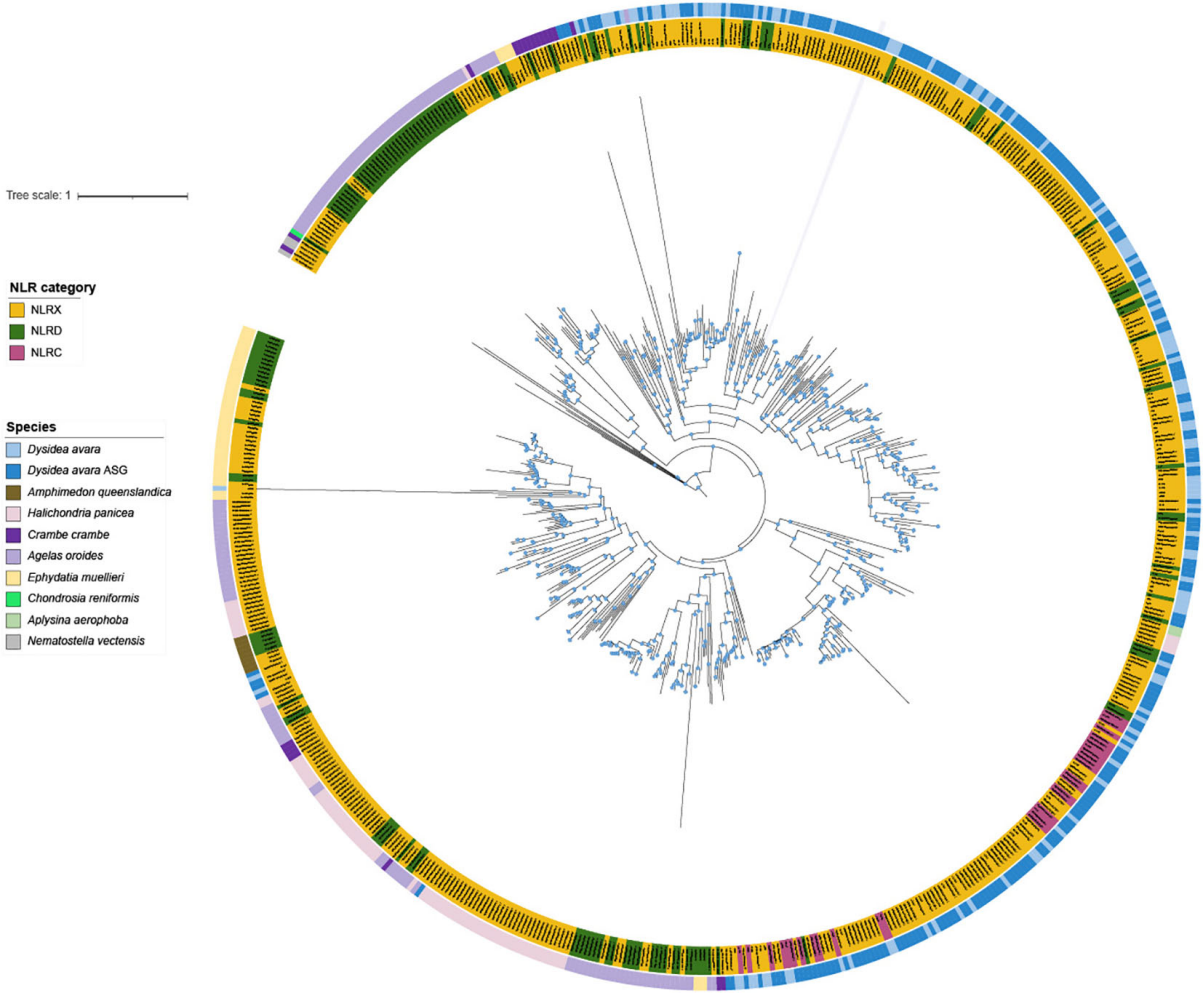
Phylogenetic analysis of NLRs from 8 sponge species based on the alignment of the NACHT domain. A phylogenetic analysis of NLRs was derived from the NACHT domain alignment in 8 sponge species, including our species (Supplementary File 1C; Supplementary File 2). As an outgroup, the NACHT domain from NLRs in the cnidarian *Nematostella vectensis* was used. The tree shows that NLRs are mainly grouped by species rather than by category. However, within the *D. avara* cluster, we see different clusters based on NLRD or NLRC category. The ML tree was constructed with GTR Bootstrap expectation model and an estimated gamma shape parameter and 100 independent searches with RAxML. NLRC, CARD–NACHT-LRR; NLRD, DEATH-NACHT-LRR; and NLRX, NACHT- LRR.

We then expanded the *bona fide* NLR search to the other 10 additional species, also including the recently available *D. avara* genome (Davar2, GCF_963678975.1) (**Supplementary Table 1**). Based on hmmer search with NACHT domain custom profile (**Supplementary Table 2A****;****Supplementary File 1**; **Supplementary File 2A**), we detected > 100 NACHT domain-containing proteins in each of the 11 sponges (**Supplementary Figure 3B**) but only a relatively small proportion (if any) of those proteins could be assigned to *bona fide* NLRs based on their domain architecture. For example, ca. 1/3 of NACHT domain-containing proteins in *D. avara* corresponded to *bona fide* NLRs, only one NLRX was detected in *O. lobullaris*, while no *bona fide* NLRs were detected at all in the hexactinellid sponge *A. vastus*, as so they were excluded from the downstream phylogenetic analysis (**Supplementary Figure 3B**). *Dysidea avara* had the largest genome span and the largest number of *bona fide* NLR genes (**Supplementary Figure 3B**). However, the number of *bona fide* NLR genes was not proportional to genome span in all species. In particular, the largest expansion relative to genome assembly span was found in *H. panicea* genome (**Supplementary Figure 3**). In all species with *bona fide* NLRs, most of them were assigned to the NLRX category, followed by NLRD category (**Supplementary Figure 3**). Looking into the phylogeny of the NACHT domain in *bona fide* NLR proteins across the different sponge species, we found that protein sequences clustered mostly by species and not by NLR category (**Figure 4**). Within the *D. avara* sequences (including those detected in both our genome and the genome generated by ASG), NLRC and NLRD were in different groups while the NLRX category appeared together with NLRC and NLRD, similar to what was described in **Figure 3**.

## 4 Discussion

This is the first study in which chromosome-level genomes of sponges were used to explore elements of innate immunity (*D. avara* genome only) and NLR expansions. Here, we generated the chromosome-level genome of the marine sponge *D. avara* and reported that this sponge contains almost the full diversity of conserved protein domains found in metazoan immune genes (**Table 1**), supporting the complex immune repertoire previously described in sponge draft genomes and transcriptomes (25, 26, 41, 64). Focusing further on NLR receptors, we observed an expansion of NACHT-containing genes in *D. avara*, detecting a large number of *bona fide* NLRs (NLRC, NLRD, NLRX) (**Figure 3**) as well as other NACHT domain-containing architectures previously described in ctenophora and cnidaria (23, 24, 65) (**Supplementary Table 9**). Finally, we reported a large expansion of NACHT-domain-containing genes in all sponges analyzed (**Supplementary Figure 3**). However, the *bona fide* NLR diversity was distinct for each species, with *D. avara, A. oroides, H. panicea, and E. muelleri* showing the largest *bona fide* NLR repertoires (**Supplementary Figure 3****;****Figure 4**).

### 4.1 Immune-related protein domains detected in *D. avara*

*Dysidea avara* chromosome-level genome comprises most of the protein domains characteristic of common metazoan immune receptors (**Table 1**), in agreement with reports in other sponge transcriptomes and genomes in this (**Supplementary Table 6**) and previous studies (26, 28, 40, 41). Some domains had larger gene expansions than others. I-set and V-set Pfam domains for instance were the most abundant in all sponge species studied here, but they were also one of the most abundant in ctenophores (65). The C-terminal LRRCT of the LRR structure was neither detected in our genome nor in any of the ASG annotated sponge genomes studied here (**Table 1****;****Supplementary Table 6**). LRRNT and LRRCT structures are mainly common in extracellular and membrane-associated LRR proteins (66). Interestingly, the ctenophores *Mnemiopsis leidyi, Bolinopsis microptera*, and *Hormiphora californensis* are also lacking the LRRCT sequence (65), indicating either that this part of the LRR structure appeared later in animal evolution or that there are different structure variants in early diverging metazoans.

The TIR domain is part of the Toll-like Receptors (TLRs) IL-1R, and Myd88 signaling gene (67–69). The canonical structure of the TLR receptor consists of the TIR domain combined with LRR as extracellular domains (16, 70, 71). However, this canonical structure is not present in sponges (26, 40, 72): the sponge homologous of the TIR domain in vertebrate TLRs, appears in combination with an extracellular immunoglobulin domain instead (40). According to that structure and homology, we identified 6 TLR-like genes in the *D. avara* genome, and the only TIR-DEATH sequence detected here is orthologous to vertebrate Myd88, according to the phylogenetic analysis conducted in a previous study (65). On the other hand, the TIR2 domain-containing genes of *D. avara* (**Table 1**), also found in the rest of studied sponge genomes (**Supplementary Table 6**) here and in previous studies (40) and previously also detected in Cnidaria (73), were homologous with those of Ctenophora, which have no signs of homology with TIR domains of TLR-like, or Myd88 structures of other metazoans (65). However, given that TIR2 domain-containing genes were overexpressed after challenging the ctenophore *Mnemiopsis leidyi* with pathogens (65), TIR2 containing proteins might also have similar functions in sponges as well.

### 4.2 An expanded Repertoire of NLRs is found in *D. avara*

The *D. avara* genome has a large expansion of NACHT domain-containing genes (126 *bona fide* NLR related genes and 128 additional genes) (**Figure 3A****;****Supplementary Tables 7**, **9**). Such large expansions of this domain have previously been found in sponge draft genomes (25) and transcriptomes (28, 41, 64), ctenophores (65) and cnidarians (23, 24). Some of NACHT-containing genes have similar domain architectures in all early diverging metazoans (e.g. NACHT-WD40; NACHT-Zu5; NACHT-Ank) (**Supplementary Table 9**) (23, 24, 65) but still with unknown function. Though animal NLRs are reported as cytosolic (31), we confirmed here the presence of transmembrane domains in NLR architectures (**Figure 2A**), suggesting putative membrane-bound NLRs, as first reported in *A. queenslandica* (25). *Bona fide* NLR genes were distributed across *D. avara* chromosomes (**Figure 2B**), but we also observed genes of the same NLR category close together in the same chromosome (i.e. in chromosome 8, we saw several copies of NLRX nearby) (**Figure 2B**). Indeed, in 2 cases, we recovered almost identical genes close together in the same chromosomes (i.e. c6_44055680.417 with c6_44055680.432; 90% similarity; c13_30875229.36 with c13_30875229.44; 96% similarity; c2_56536150.10 with c2_56536150.14; 96.2% similarity), all belonging to NLRX category. Hence, we can hypothesize that NLR diversity in *D. avara* responds to gene duplication events. On another note, NLRX was also in close proximity with either NLRD or NLRC in most *D. avara* chromosomes (**Figure 2B**). This was also observed in the phylogenetic analysis of *D. avara* NLRs, in which NLRXs formed distinct clusters within either the NLRD or NLRC group (**Figure 3**). From the above, it is suggested that NLRD and NLC categories are true paralogues derived from NLRX. NLRXs have already been detected in Bacteria (as NB-ARC analogs), Fungi and protists (74) and they have been suggested as the ancestral core of NLR gene family, while the N-terminal DEATH/CARD was a later addition in the evolution of metazoan NLRs, providing the advantage of modulating signaling pathways of immunity and apoptosis (75).

### 4.3 NLR evolution in porifera

Our analysis of NLR diversity in additional chromosome-level sponge genomes revealed a high number of NACHT-domain containing genes in all species analyzed. The overall large expansion of immune gene families, including NLR-encoding genes, is a common characteristic of non-vertebrates (i.e. *Hydra*, sea anemone, sea urchin, amphioxus (76–79)), and this can be explained by the fact that they lack an adaptive immune system, and as such they rely on expansions of innate immune sensors to maintain effective immune defenses. In all cases, the NLRX category was the most expanded, followed by the NLRD category. Based on our pipeline, most of the species analyzed lacked NLRCs, except for *D. avara* (20 genes), *H. panicea* (1 gene), and *C. crambe* (1 gene). In a previous NLR analysis on the draft genome of *A. queenslandica* which included different gene models, Yuen et al. (25) identified 15 NLRC within a clade that contained NLRX and NLRD genes, with the latter group dominating *A. queenslandica* NLR diversity. In fact, the DEATH domain is part of apoptotic and immune regulators in basal metazoans, including sponges, and evolved earlier than the CARD domain, with the latter to be derived from the DEATH family (80). On the other hand, the CARD domain is more commonly found on the NLRs of bilaterians which make part of their inflammasome (81). This evolutionary trajectory agrees with the findings of a larger expansion of NLRD *vs* NLRC categories in our study (**Figure 4****;****Supplementary Figure 3B****;****Supplementary Table 7**), but also with reports in other marine invertebrates. Indeed, very few CARD domains were identified in the sea urchin genomes, and all the CARD-derived NLR functions of vertebrates were replaced by DEATH domain gene families, which dominate the genomes of sea urchin and amphioxus (76, 82). Under this evolutionary context, NLRs in Porifera, including NLRD and NLRC, were found in the most primitive position in the evolution of metazoan NLRs, which further expanded to either exclusively NLRD in certain invertebrates, or NLRC across most invertebrates and vertebrates (83). Overall, the presence of NLRC and NLRD already in sponge genomes suggests that these ancestral genes were already present in the last common ancestor of metazoans, making Porifera central to the origin of NLR evolution.

### 4.4 NLR repertoire in sponges is species-specific

The analysis of NLR categories revealed differences in the diversity of *bona fide* NLR repertoire among Porifera. The higher number of NACHT-domain containing genes and *bona*.

*fide* NLR genes recovered from the ASG *D. avara* genome in comparison to our genome.

(**Supplementary Table 1**; **Supplementary Figure 3**) are likely explained by the pipelines used.

for genome assembly and gene model predictions. Yet, both genomes support that *D. avara* has.

the largest number of *bona fide* NLRs (**Supplementary Figure 3**) and immune genes (**Table 1**; **Supplementary Table 6**) of all demosponges, pointing to a true biological difference. Following *D. avara*, *A. oroides* and *H. panicea* also presented large *bona fide* NLR expansions. Previously, Posadas et al. (64), studied the NLR repertoire in 16 sponge transcriptomes, identifying the largest number of NLRs in *D. avara* transcriptome (50 genes) as well. The variation in gene expansion and molecular divergence of NLRs among the different sponge species (**Figure 4**; **Supplementary Figure 3**) suggests different evolutionary trajectories to distinct selective pressures (84). The reduced repertoire of *bona fide* NLR compared to NACHT-domain containing genes may be explained by differences in the evolutionary history across species, divergent architectures of NACHT domain-containing genes and/or highly divergent LRR domains that could be missed in our analytical approach. Pathogen presence is a crucial selective agent in immune evolution, though here we analyzed species that occur in sympatry and at least in recent evolutionary times had likely encountered similar bacteria in the water while filter feeding. The maintenance of symbiotic host-microbe interactions can also dictate immune repertoires. Precisely, low microbial abundant (LMA) sponges, like *D. avara*, were suggested to have a larger expansion and higher diversity of NLR repertoire compared to HMAs (25, 85–87). For instance, the LMA sponge *Stylissa carteri* has a more expanded repertoire of immune domains, including the NACHT domain, than the HMA *Xestospongia testudinaria* (88). Large expansions in immune genes can also be correlated with variations in functions, or with allowing high expression levels (89, 90). Interestingly, some NLR genes were overexpressed while others were downregulated in the LMA *D. avara* while in contact with microbial-associated molecular patterns (a cocktail of bacterial lipopolysaccharide and peptidoglycan) (26) and non-symbiotic bacteria (42). On the other hand, NLRs did not participate at all during similar bacterial exposures in the HMA sponge *Aplysina aerophoba* (42). This means that NLRs could have different functions in different species and that the function may be related to the LMA-HMA status. However, we did not identify a clear pattern of HMA-LMA status on NLR expansion in our study (**Supplementary Figure 3**). Based on the species-specific trajectories suggested by our phylogenetic analysis, we propose that the evolution of NLR reflects species-specific traits, although functional convergence may rise from the LMA-HMA status.

## 5 Conclusion

The generation of a high-quality, chromosome-level genome for *D. avara* has enabled us to make a comprehensive investigation into the immune repertoire of this sponge, with a particular focus on NOD-like Receptors (NLRs). Our findings revealed a significant expansion of immune-related protein domains, including a notable presence of distinct NLR categories within *D. avara*. NLRX had the largest expansion, followed by NLRD, while the NLRC category was represented only by a few genes. Most likely, NLRs in sponges were diversified by NLRX. The species-specific grouping of NLRs across sponge genomes highlights that NLRs were diversified in sponges in order to respond to lineage-specific evolutionary patterns related to their immunity. This study provides valuable genomic resources, protocols, and novel insights into the early evolution of animal immune systems, reinforcing the importance of Porifera as a key group for understanding the origins and diversification of innate immunity.

## Data Availability

The datasets presented in this study can be found in online repositories. The names of the repository/repositories and accession number(s) can be found in the article/**Supplementary Material**.
