# Supplementary material for: NOD-like receptor repertoire in the chromosome-level genome of the demosponge Dysidea avara (Schmidt, 1862)
Source: Front Immunol. 2026 Feb 3;17:1725140. doi: 10.3389/fimmu.2026.1725140 (PMC12909245; doi:10.3389/fimmu.2026.1725140)
Supplement: Supplementary file 2 [file DataSheet2.pdf]

Supplementary File 2A. Consensus sequence from NACHT alignment from different taxa retrieved from NCBI to construct the NACHT hmmerProfile.

```
>NACHT_domain_alignmnent
XXEPEXEXPAEHAXYPSHLIEKIRXXYXSQEDAIIPXIDXXXXNLPLVLFGDQGTGKTAVISRIVARI
WCDXXXFZLVLRDIFLRLIXXIVXKEKXXXXXXXXXXXGXKXTKEVXXXXXXXSDJSDLFTPHLESXX
XXEPRVVLXEGEAYLDFIDNGSSIDWLPNYLAPNVRLILTTRPEVKRLSLFGARSLLRNESFLELSPL
PGIGKTTLCRKLLRDXXKWASGQLXKXXXRFCGXSXSSFYGAFPPRFELLLLLRDAIKWKSCRXINSX
SNXPXAIXDLLLPDYTXPEEXYXEXXXDXFXQFLKENPXESKVLLILDLLSNNLEGLDELKYFXXXXQ
TELCTDXXPEXSXPXLALFLSLLRRKALLPNASVJVTIRSQESRPEAXSQGLRQIRSXPXXYXDYRTJ
EILGFXSDLQEEEDXKEYIRKLRLNQPYCGYFSEEXGXXXXXXXXXHLAKKLXSXXZXNSLLAXXLCX
XXXXXXSVPSTTTLLFLXLLCXIIXLLEDJXSGXPLXXVXLLXTLTSLYVEIVLLXXXXFLRRREIXX
NHGKGLVXXGXDLLSAYXKEFXXLKXXXZXXLLGLGXXXLALEGLXXXLKXXXKLIFSEEDLDDFLXDL
XLLESGXFFGFLSLLSRLRQDAGXXXXKGXSEXXPXXRVXAYAFFHKSFQEFFAARYLASLLXSXELS
DPELLKDCLSXXXVLXDLXSTGELXGKXXPGRLXVVLKFLAGXXXLXXSXXXSXXESXFLXXXLLLLX
EAJXEZITXXLVXEXGRXXRRXXEXXEPALLGYDLXSECLYESQELFTDXLTAQS
```

Supplementary File 2B. Consensus sequence from alignment of NLR sequences from D. avara. Sequences were retrieved from the automatic genome annotation and hmmer search.

```
 >NACHT_domain_alignmnent_D_avara
LKRXXXLRYINTRFTVDSGXXXXXXDEDPWPKLPNQLPIPKYFEKXKENSIXDVIXNTPLALVHHXJK
XXXKKXKGVRTKDXNXXXXXXXBPXXXKELTEIAKLTXTGXXFXXXXXXXXXXXXXXXXXDIDSILSK
KSSPKXXKLDSSHSLLELLLXSSTXSHNKQNMKSKVTKDLSDIFAPLEKSSNTSDXESXXXXXXSPCP
XLRTINNNLIEGAPXXXXXXXXXXXXXXXXAGIGKTTLDYNALDAKEICYQWARKRDGKLLQKFKTTH
PGLVLLLPLGSNNVKPADTERDPAVQXXXKITSLEDLLQENREIKPAVMEPTTPITTPIXLFCKGDFL
CHYTKAEEIAEACYEYLKKXGGKDVTFJLDGYDELPEEELRKXXXXSRDXXSFIADLIERXCKSXGXV
LPKCTJVVTSRPHASSVSLRQXXXXQAVFDRXRVEILGFTEEEREEYIKQSLQKXKGQENGPQKVKEL
TKYLTPRYSSNWKKIGIHLNVEKGQQHPTINSLCYIPFNMVILVFLFXXKQGEXNXIPLPXNSTSTEL
YKLFICLTICRHLAKTSGXPLLHVCCSSVAAANXAEDNTITDLADLPEPYNKIVQQKLSKLALKGLNN
WYSVREEDKXKKVKVKVKYKNKKLVFTLEEIXXXDXXXKAAXCPDIEAIPGDAINGFGLLQAVHEHFG
LTGKGXPTKSFNFLHLSIQEFLAAYYIISTLPPDEQLRLLRGSXXXXKEKFLVTGYGDDDWSXXXXXD
TDXKKXXTXXDEPSSKSDRHSNMFIFYVGLALTXXXBXXXXAXXXXXXXKGQRSSSSFKQFPLSRX
```

Supplementary File 2C. Consensus sequence from alignment of NLR sequences from all sponge species included in phylogenetic analysis. Sequences were retrieved from hmmer search based on the constructed NACHT hmmer profile.

```
>NACHT_domain_alignmnent_all_Porifera
LILIEGAPGEYYKVLVVVXXXXXXXGXXXXXIGKTTLAKEICYKWAXKRKGXXEQQTIPGSLEMXXXT
XXXXXXXXXFGGKLLLQDFDLVLLLPLRDFPNMVVQKXXXXXXPAKKXXSLEEDLXRYLLDQLFIGGE
TXXXXAXQXATEIAEAVYEYSPVLLFKKSGGTKGXXXXVLFILDGYCRKYLDELPEECQXXXXXXJXL
RXXIEKDXXNAEGSFFAFNDLVIXPXXSKGDMSEVTLLLNDLYTIALQMRHPKATVVVTSRCTPHASA
PEQISQGYNLYKHFVGGNVLHLKGLCDVQDYIRKXLKLHQFCNGXXXXXXXXXXXXXXXXXXXXXXXX
XXXXXXXXXXXXXKXXSXXXXXXXXXXXXXXXXXXXXXXXXXXDZXXXJXXZXXXXXXXXJXXXXXTX
XXXXXXXXLXXLCFLXLCPLCLVDRQISQRIEILEEDDNVEEDDGFTKEEIKEQVLQYIEQAKKLKSS
VKDD
```
