# Supplementary material for: NOD-like receptor repertoire in the chromosome-level genome of the demosponge Dysidea avara (Schmidt, 1862)
Source: Front Immunol. 2026 Feb 3;17:1725140. doi: 10.3389/fimmu.2026.1725140 (PMC12909245; doi:10.3389/fimmu.2026.1725140)
Supplement: Supplementary file 3 [file DataSheet3.pdf]

Supplementary Material

Supplementary Tables

Supplementary Table 1. Sponge species included in the immune Pfam domains search and NLR phylogenetic analysis

Species	Class	Version/ID	Genome span (Mb)	Source
Agelas oroides	Demospongiae	GCA_949730485.1	261	ASG project
Amphimedon queenslandica	Demospongiae	GCA_000090795.2	164	Srivastava et al. 2010 (1)
Aplysina aerophoba	Demospongiae	GCA_949841015.1	159	ASG project (2) https://doi.org/10.12688/wellcomeopenres.24098.1
Aphrocallistes vastus	Hexactinellida	Avas.v1.29	80	Francis et al. 2023 (3)
Chondrosia reniformis	Demospongiae	GCA_947172415.1	117	ASG project (4) https://doi.org/10.12688/wellcomeopenres.24166.1
Crambe crambe	Demospongiae	GCA_963924555.1	143	ASG project (5) https://doi.org/10.12688/wellcomeopenres.24154.1
D. avara	Demospongiae	GCA_963678975.2	551	ASG project
Ephydatia muelleri	Demospongiae	Emu_v1	323	Kenny et al. 2020 (6)
Halichondria panicea	Demospongiae	GCA_963675165.1	131	ASG project
Oscarella lobularis	Homoscleromorpha	GCA_947507565.1	65	ASG project (7) https://doi.org/10.12688/wellcomeopenres.24187.1

Supplementary Table 2A. Sequences ID retrieved from NCBI to construct the hmmer profile for NACHT domain used in phylogeny.

NCBI_IDs	Species	Taxonomy
Adi_NLRb_XP_015761610.1	*Acropora digitifera*	Cnidaria; Anthozoa
Ame_NACHT_XP_026296342.1	*Apis mellifera*	Ecdysozoa; Arthropoda
Ami_NLR_XP_044178921.1	*Acropora millepora*	Cnidaria; Anthozoa
Ami_NLRb_XP_044176601.1	*Acropora millepora*	Cnidaria; Anthozoa
Ami_NLRc_XP_044171927.1	*Acropora millepora*	Cnidaria; Anthozoa
Aque_NACHT_XP_019856992.1	*Amphimedon queensladica*	Porifera; Demospongiae
Cgi_NLR_UVD39213.1	*Crassostrea gigas*	Spiralia; Lophotrochozoa; Mollusca
Gb_NACHT_CAI8003661.1	*Geodia barretti*	Porifera; Demospongiae
Hsa_NLR_XP_054199222.1	*Homo sapiens*	Chordata; Craniata
Hsa_NLR_f_KAI4047375.1	*Homo sapiens*	Chordata; Craniata
Hsa_NLRb_NP_001269287.1	*Homo sapiens*	Chordata; Craniata
Hsa_NLRc_NP_849172.2	*Homo sapiens*	Chordata; Craniata
Hsa_NLRd_KAI4085643.1	*Homo sapiens*	Chordata; Craniata
Hsa_NLRe_KAI4055029.1	*Homo sapiens*	Chordata; Craniata
Hvu_NACHT_XP_047131535.1	*Hydra vulgaris*	Cnidaria; Hydrozoa
Hvu_NLR_XP_047131182.1	*Hydra vulgaris*	Cnidaria; Hydrozoa
Hvu_NLR_ADU79234.1	*Hydra vulgaris*	Cnidaria; Hydrozoa

Nme_NACHT_XP_048579888.1	*Nematostella vectensis*	Cnidaria; Anthozoa
Nve_NLR_XP_048579698.1	*Nematostella vectensis*	Cnidaria; Anthozoa
Nve_NLR_C_XP_048575673.1	*Nematostella vectensis*	Cnidaria; Anthozoa
Nve_NLR_XP_048578881.1	*Nematostella vectensis*	Cnidaria; Anthozoa
Omi_NACHT_KAI6649186.1	*Oopsacas minuta*	Porifera; Hexactinellida
Pda_NLR_XP_027052631.1	*Pocillopora damicornis*	Cnidaria; Anthozoa
Sci_NLR_CAH0815927.1	*Sycon ciliatum*	Porifera; Calcarea
Spu_NACHT_XP_011660817.2	*Strongylocentrotus purpuratus*	Echinodermata; Eleutherozoa
Spu_NLR_XP_030838420.1	*Strongylocentrotus purpuratus*	Echinodermata; Eleutherozoa
Spu_NLRb_XP_011677849.2	*Strongylocentrotus purpuratus*	Echinodermata; Eleutherozoa
Trichoplax_NACHTWD_RDD39150.1	*Trichoplax sp.*	Placozoa; Uniplacotomia

Supplementary Table 2B. NLR sequences extracted from *Dysidea avara* genome for the phylogeny of NLR categories within the species (Figure 3).

**ID_seuquence of *D. avara***
c11.240
c11.407
c11.585
c11.586
c11.588
c11.668
c11.885
c11.1306
c11.1346
c11.1370
c11.1393
c11.1422

c11.1462
c11.1467
c11.1475
c12.225
c12.278
c12.1035
c12.1128
c12.1514
c12.1550
c12.1555
c13.30
c13.36
c13.44
c13.102
c13.417
c13.486
c13.595
c13.987
c13.1659
c14.399
c14.400
c14.411
c14.427
c14.479
c14.529
c14.1151
c15.6
c15.47
c15.93
c15.394
c15.472
c15.780
c15.994
c15.1096
c15.1203
c15.1385
c15.1394
c15.1595
c5.375
c5.1282

c6.77
c6.109
c6.126
c6.151
c6.157
c6.294
c6.417
c6.432
c6.438
c6.733
c6.739
c6.877
c6.878
c6.888
c6.1323
c6.1338
c6.1389
c6.1648
c6.1931
c7.266
c7.273
c7.279
c7.283
c7.296
c7.299
c7.337
c7.350
c7.366
c7.368
c7.379
c7.457
c7.588
c7.649
c7.712
c7.740
c7.1153
c7.1419
c7.1832
c8.26
c8.32
c8.33

c8.37
c8.41
c8.61
c8.64
c8.69
c8.128
c8.142
c8.170
c8.187
c8.705
c8.1191
c8.1237
c8.1789
c8.1791
c8.1819
c8.1820
c8.2188
c9.592
c10.432
c10.555
c10.648
c10.708
c10.954
c1.948
c1.1751
c1.1824
c1.2148
c1.2403
c1.2404
c2.10
c2.14
c2.48
c2.51
c2.56
c2.100
c2.205
c2.711
c2.1017
c2.1020
c2.1050

c2.1051
c2.1462
c2.1463
c2.1908
c3.966
c3.1149
c3.1164
c3.1200
c3.1567
c3.1610
c3.1773
c3.1889
c4.226
c4.334
c4.361

Supplementary Table 2C. NLR sequences extracted from all sponge species used for the phylogeny of NLR categories among species (Figure 4).

ID_sequence	Sponge Species
BRAKEROCXP00000000033.1	*C. crambe*
BRAKEROCXP00000000287.1	*C. crambe*
BRAKEROCXP00000000401.1	*C. crambe*
BRAKEROCXP00000000669.1	*C. crambe*
BRAKEROCXP00000002915.1	*C. crambe*
BRAKEROCXP00000004213.1	*C. crambe*
BRAKEROCXP00000004475.1	*C. crambe*
BRAKEROCXP00000006188.1	*C. crambe*
BRAKEROCXP00000009728.1	*C. crambe*
BRAKEROCXP00000010030.1	*C. crambe*
BRAKEROCXP00000012643.1	*C. crambe*
BRAKEROCXP00000013116.1	*C. crambe*
BRAKEROCXP00000015133.1	*C. crambe*
BRAKEROCXP00000015136.1	*C. crambe*
BRAKEROCXP00000015729.1	*C. crambe*
BRAKEROCXP00000015860.1	*C. crambe*
BRAKEROCXP00000016112.1	*C. crambe*
BRAKEROCXP00000016291.1	*C. crambe*
BRAKEROCXP00000016457.1	*C. crambe*
BRAKEROCXP00000017357.1	*C. crambe*

BRAKEROCXP00000017641.1	*C. crambe*
Em0001g2538a	*E. muelleri*
Em0001g2558a	*E. muelleri*
Em0001g2602a	*E. muelleri*
Em0001g2631a	*E. muelleri*
Em0001g2856a	*E. muelleri*
Em0001g2861a	*E. muelleri*
Em0001g2863a	*E. muelleri*
Em0001g2875a	*E. muelleri*
Em0001g2881a	*E. muelleri*
Em0001g2891a	*E. muelleri*
Em0001g2898a	*E. muelleri*
Em0001g2922a	*E. muelleri*
Em0001g2927a	*E. muelleri*
Em0001g2931a	*E. muelleri*
Em0001g2935a	*E. muelleri*
Em0001g2940a	*E. muelleri*
Em0001g2943a	*E. muelleri*
Em0001g3749a	*E. muelleri*
Em0001g808a	*E. muelleri*
Em0002g1077a	*E. muelleri*
Em0002g1099a	*E. muelleri*
Em0002g654a	*E. muelleri*
Em0002g656a	*E. muelleri*
Em0002g660a	*E. muelleri*
Em0002g666a	*E. muelleri*
Em0002g668a	*E. muelleri*
Em0002g689a	*E. muelleri*
Em0002g695a	*E. muelleri*
Em0002g716a	*E. muelleri*
Em0002g718a	*E. muelleri*
Em0002g725a	*E. muelleri*
Em0002g773a	*E. muelleri*
Em0002g784a	*E. muelleri*
Em0002g786a	*E. muelleri*
Em0002g787a	*E. muelleri*
Em0003g1730a	*E. muelleri*
Em0005g904a	*E. muelleri*
Em0005g905a	*E. muelleri*
Em0005g910a	*E. muelleri*

Em0006g1336a	*E. muelleri*
Em0014g368a	*E. muelleri*
Em0016g313a	*E. muelleri*
Em0016g328a	*E. muelleri*
Em0018g359a	*E. muelleri*
ENSFPCP00005001793.1	*H. panicea*
ENSFPCP00005002939.1	*H. panicea*
ENSFPCP00005002947.1	*H. panicea*
ENSFPCP00005008063.1	*H. panicea*
ENSFPCP00005008068.1	*H. panicea*
ENSFPCP00005008074.1	*H. panicea*
ENSFPCP00005008595.1	*H. panicea*
ENSFPCP00005011219.1	*H. panicea*
ENSFPCP00005011220.1	*H. panicea*
ENSFPCP00005012321.1	*H. panicea*
ENSFPCP00005012471.1	*H. panicea*
ENSFPCP00005012537.1	*H. panicea*
ENSFPCP00005012705.1	*H. panicea*
ENSFPCP00005013132.1	*H. panicea*
ENSFPCP00005013169.1	*H. panicea*
ENSFPCP00005013377.1	*H. panicea*
ENSFPCP00005013638.1	*H. panicea*
ENSFPCP00005013698.1	*H. panicea*
ENSFPCP00005013738.1	*H. panicea*
ENSFPCP00005016578.1	*H. panicea*
ENSFPCP00005016598.1	*H. panicea*
ENSFPCP00005017775.1	*H. panicea*
ENSFPCP00005018537.1	*H. panicea*
ENSFPCP00005020408.1	*H. panicea*
ENSFPCP00005022275.1	*H. panicea*
ENSFPCP00005022642.1	*H. panicea*
ENSFPCP00005026286.1	*H. panicea*
ENSFPCP00005026292.1	*H. panicea*
ENSFPCP00005026992.1	*H. panicea*
ENSFPCP00005026998.1	*H. panicea*
ENSFPCP00005027004.1	*H. panicea*
ENSFPCP00005030990.1	*H. panicea*
ENSFPCP00005030999.1	*H. panicea*
ENSFPCP00005031003.1	*H. panicea*
ENSFPCP00005031465.1	*H. panicea*
ENSFPCP00005031471.1	*H. panicea*

ENSFPCP00005031475.1	*H. panicea*
ENSFPCP00005033127.1	*H. panicea*
ENSFPCP00005033175.1	*H. panicea*
ENSFPCP00005033182.1	*H. panicea*
ENSFPCP00005033541.1	*H. panicea*
ENSFPCP00005034456.1	*H. panicea*
ENSFPCP00005035578.1	*H. panicea*
ENSFPCP00005035579.1	*H. panicea*
ENSFPCP00005035590.1	*H. panicea*
ENSFPCP00005035712.1	*H. panicea*
ENSFPCP00005035717.1	*H. panicea*
ENSFPCP00005035759.1	*H. panicea*
ENSFPCP00005036354.1	*H. panicea*
ENSFPCP00005036425.1	*H. panicea*
ENSFPCP00005036499.1	*H. panicea*
ENSFPCP00005036842.1	*H. panicea*
ENSFPCP00005037091.1	*H. panicea*
ENSFPCP00005037102.1	*H. panicea*
ENSFPCP00005037147.1	*H. panicea*
ENSFPCP00005037175.1	*H. panicea*
ENSFPCP00005037853.1	*H. panicea*
ENSFPCP00005037889.1	*H. panicea*
ENSFPCP00005037950.1	*H. panicea*
ENSFPCP00005037951.1	*H. panicea*
ENSFPCP00005037961.1	*H. panicea*
ENSFPCP00005037970.1	*H. panicea*
ENSFPCP00005037972.1	*H. panicea*
ENSFPCP00005038037.1	*H. panicea*
ENSFPCP00005038039.1	*H. panicea*
ENSFPCP00005038219.1	*H. panicea*
ENSFPCP00005038484.1	*H. panicea*
ENSFPCP00005038702.1	*H. panicea*
ENSFPCP00005038846.1	*H. panicea*
ENSFPCP00005038850.1	*H. panicea*
ENSFPCP00005039188.1	*H. panicea*
ENSFPCP00005039199.1	*H. panicea*
ENSFPCP00005039308.1	*H. panicea*
ENSFPCP00005039361.1	*H. panicea*
ENSFPCP00005039782.1	*H. panicea*
ENSFPCP00005039839.1	*H. panicea*

ENSFPCP00005040510.1	*H. panicea*
ENSLSGP00005000143.1	*A. oroides*
ENSLSGP00005000154.1	*A. oroides*
ENSLSGP00005000685.1	*A. oroides*
ENSLSGP00005000744.1	*A. oroides*
ENSLSGP00005000832.1	*A. oroides*
ENSLSGP00005001198.1	*A. oroides*
ENSLSGP00005002112.1	*A. oroides*
ENSLSGP00005002134.1	*A. oroides*
ENSLSGP00005002429.1	*A. oroides*
ENSLSGP00005002432.1	*A. oroides*
ENSLSGP00005002786.1	*A. oroides*
ENSLSGP00005002846.1	*A. oroides*
ENSLSGP00005003045.1	*A. oroides*
ENSLSGP00005003276.1	*A. oroides*
ENSLSGP00005004692.1	*A. oroides*
ENSLSGP00005004716.1	*A. oroides*
ENSLSGP00005004987.1	*A. oroides*
ENSLSGP00005004993.1	*A. oroides*
ENSLSGP00005005082.1	*A. oroides*
ENSLSGP00005005321.1	*A. oroides*
ENSLSGP00005005580.1	*A. oroides*
ENSLSGP00005005640.1	*A. oroides*
ENSLSGP00005009887.1	*A. oroides*
ENSLSGP00005011386.1	*A. oroides*
ENSLSGP00005011742.1	*A. oroides*
ENSLSGP00005012301.1	*A. oroides*
ENSLSGP00005012307.1	*A. oroides*
ENSLSGP00005012317.1	*A. oroides*
ENSLSGP00005012324.1	*A. oroides*
ENSLSGP00005012435.1	*A. oroides*
ENSLSGP00005012722.1	*A. oroides*
ENSLSGP00005012995.1	*A. oroides*
ENSLSGP00005013004.1	*A. oroides*
ENSLSGP00005013008.1	*A. oroides*
ENSLSGP00005013826.1	*A. oroides*
ENSLSGP00005013916.1	*A. oroides*
ENSLSGP00005014208.1	*A. oroides*
ENSLSGP00005014404.1	*A. oroides*
ENSLSGP00005014419.1	*A. oroides*
ENSLSGP00005014902.1	*A. oroides*

ENSLSGP00005016047.1	*A. oroides*
ENSLSGP00005016769.1	*A. oroides*
ENSLSGP00005017327.1	*A. oroides*
ENSLSGP00005017817.1	*A. oroides*
ENSLSGP00005017822.1	*A. oroides*
ENSLSGP00005018359.1	*A. oroides*
ENSLSGP00005018674.1	*A. oroides*
ENSLSGP00005018691.1	*A. oroides*
ENSLSGP00005019044.1	*A. oroides*
ENSLSGP00005020055.1	*A. oroides*
ENSLSGP00005020103.1	*A. oroides*
ENSLSGP00005021194.1	*A. oroides*
ENSLSGP00005021208.1	*A. oroides*
ENSLSGP00005022306.1	*A. oroides*
ENSLSGP00005022312.1	*A. oroides*
ENSLSGP00005022331.1	*A. oroides*
ENSLSGP00005022338.1	*A. oroides*
ENSLSGP00005022789.1	*A. oroides*
ENSLSGP00005022808.1	*A. oroides*
ENSLSGP00005023106.1	*A. oroides*
ENSLSGP00005023281.1	*A. oroides*
ENSLSGP00005023287.1	*A. oroides*
ENSLSGP00005023306.1	*A. oroides*
ENSLSGP00005023328.1	*A. oroides*
ENSLSGP00005023567.1	*A. oroides*
ENSLSGP00005023714.1	*A. oroides*
ENSLSGP00005023735.1	*A. oroides*
ENSLSGP00005023742.1	*A. oroides*
ENSLSGP00005024046.1	*A. oroides*
ENSLSGP00005024290.1	*A. oroides*
ENSLSGP00005024445.1	*A. oroides*
ENSLSGP00005024682.1	*A. oroides*
ENSLSGP00005024683.1	*A. oroides*
ENSLSGP00005024917.1	*A. oroides*
ENSLSGP00005025087.1	*A. oroides*
ENSLSGP00005025207.1	*A. oroides*
ENSLSGP00005025218.1	*A. oroides*
ENSLSGP00005025656.1	*A. oroides*
ENSLSGP00005025711.1	*A. oroides*
ENSLSGP00005025891.1	*A. oroides*

ENSLSGP00005026219.1	*A. oroides*
ENSLSGP00005026263.1	*A. oroides*
ENSLSGP00005026430.1	*A. oroides*
ENSLSGP00005026561.1	*A. oroides*
ENSLSGP00005026576.1	*A. oroides*
ENSLSGP00005027287.1	*A. oroides*
ENSLSGP00005027303.1	*A. oroides*
ENSLSGP00005028353.1	*A. oroides*
ENSLSGP00005028360.1	*A. oroides*
ENSLSGP00005028460.1	*A. oroides*
ENSLSGP00005028720.1	*A. oroides*
ENSLSGP00005028974.1	*A. oroides*
ENSLSGP00005029057.1	*A. oroides*
ENSLSGP00005029072.1	*A. oroides*
ENSLSGP00005029445.1	*A. oroides*
ENSLSGP00005029936.1	*A. oroides*
ENSLSGP00005029951.1	*A. oroides*
ENSLSGP00005029975.1	*A. oroides*
ENSLSGP00005029980.1	*A. oroides*
ENSLSGP00005030381.1	*A. oroides*
ENSLSGP00005030774.1	*A. oroides*
ENSLSGP00005030805.1	*A. oroides*
ENSLSGP00005031139.1	*A. oroides*
ENSLSGP00005031287.1	*A. oroides*
ENSLSGP00005031299.1	*A. oroides*
ENSLSGP00005031399.1	*A. oroides*
ENSLSGP00005031439.1	*A. oroides*
ENSLSGP00005031511.1	*A. oroides*
ENSLSGP00005031524.1	*A. oroides*
ENSLSGP00005031715.1	*A. oroides*
ENSLSGP00005031740.1	*A. oroides*
ENSLSGP00005031746.1	*A. oroides*
ENSLSGP00005032331.1	*A. oroides*
ENSLSGP00005032344.1	*A. oroides*
ENSLSGP00005032349.1	*A. oroides*
ENSLSGP00005032358.1	*A. oroides*
ENSLSGP00005032497.1	*A. oroides*
ENSLSGP00005032511.1	*A. oroides*
ENSLSGP00005032611.1	*A. oroides*
ENSLSGP00005032716.1	*A. oroides*
ENSLSGP00005032924.1	*A. oroides*

ENSLSGP00005032935.1	*A. oroides*
ENSLSGP00005032950.1	*A. oroides*
ENSLSGP00005033169.1	*A. oroides*
ENSLSGP00005033551.1	*A. oroides*
ENSLSGP00005033617.1	*A. oroides*
ENSLSGP00005033714.1	*A. oroides*
ENSLSGP00005033721.1	*A. oroides*
ENSLSGP00005033729.1	*A. oroides*
ENSOMHP00030018445.1	*A. aerophoba*
ENSOMHP00030020115.1	*A. aerophoba*
ENSOUGP00010000537.1	*D. avara2*
ENSOUGP00010000920.1	*D. avara2*
ENSOUGP00010001002.1	*D. avara2*
ENSOUGP00010001143.1	*D. avara2*
ENSOUGP00010001334.1	*D. avara2*
ENSOUGP00010001489.1	*D. avara2*
ENSOUGP00010001544.1	*D. avara2*
ENSOUGP00010001658.1	*D. avara2*
ENSOUGP00010001813.1	*D. avara2*
ENSOUGP00010001866.1	*D. avara2*
ENSOUGP00010002156.1	*D. avara2*
ENSOUGP00010002165.1	*D. avara2*
ENSOUGP00010002297.1	*D. avara2*
ENSOUGP00010002518.1	*D. avara2*
ENSOUGP00010002604.1	*D. avara2*
ENSOUGP00010002614.1	*D. avara2*
ENSOUGP00010002773.1	*D. avara2*
ENSOUGP00010004077.1	*D. avara2*
ENSOUGP00010004591.1	*D. avara2*
ENSOUGP00010004630.1	*D. avara2*
ENSOUGP00010005298.1	*D. avara2*
ENSOUGP00010005356.1	*D. avara2*
ENSOUGP00010005874.1	*D. avara2*
ENSOUGP00010005949.1	*D. avara2*
ENSOUGP00010005953.1	*D. avara2*
ENSOUGP00010006221.1	*D. avara2*
ENSOUGP00010006930.1	*D. avara2*
ENSOUGP00010006981.1	*D. avara2*
ENSOUGP00010006986.1	*D. avara2*
ENSOUGP00010007090.1	*D. avara2*

ENSOUGP00010007361.1	*D. avara2*
ENSOUGP00010007378.1	*D. avara2*
ENSOUGP00010007900.1	*D. avara2*
ENSOUGP00010007906.1	*D. avara2*
ENSOUGP00010008223.1	*D. avara2*
ENSOUGP00010008260.1	*D. avara2*
ENSOUGP00010008365.1	*D. avara2*
ENSOUGP00010008375.1	*D. avara2*
ENSOUGP00010009273.1	*D. avara2*
ENSOUGP00010009285.1	*D. avara2*
ENSOUGP00010009289.1	*D. avara2*
ENSOUGP00010009432.1	*D. avara2*
ENSOUGP00010009509.1	*D. avara2*
ENSOUGP00010010043.1	*D. avara2*
ENSOUGP00010010368.1	*D. avara2*
ENSOUGP00010011004.1	*D. avara2*
ENSOUGP00010011280.1	*D. avara2*
ENSOUGP00010011283.1	*D. avara2*
ENSOUGP00010011286.1	*D. avara2*
ENSOUGP00010012543.1	*D. avara2*
ENSOUGP00010013259.1	*D. avara2*
ENSOUGP00010013418.1	*D. avara2*
ENSOUGP00010013617.1	*D. avara2*
ENSOUGP00010013648.1	*D. avara2*
ENSOUGP00010013657.1	*D. avara2*
ENSOUGP00010013762.1	*D. avara2*
ENSOUGP00010013881.1	*D. avara2*
ENSOUGP00010013897.1	*D. avara2*
ENSOUGP00010013898.1	*D. avara2*
ENSOUGP00010013920.1	*D. avara2*
ENSOUGP00010013928.1	*D. avara2*
ENSOUGP00010013944.1	*D. avara2*
ENSOUGP00010013951.1	*D. avara2*
ENSOUGP00010014938.1	*D. avara2*
ENSOUGP00010014948.1	*D. avara2*
ENSOUGP00010014959.1	*D. avara2*
ENSOUGP00010014965.1	*D. avara2*
ENSOUGP00010014980.1	*D. avara2*
ENSOUGP00010014987.1	*D. avara2*
ENSOUGP00010014999.1	*D. avara2*
ENSOUGP00010015143.1	*D. avara2*

ENSOUGP00010015781.1	*D. avara2*
ENSOUGP00010015787.1	*D. avara2*
ENSOUGP00010015859.1	*D. avara2*
ENSOUGP00010016783.1	*D. avara2*
ENSOUGP00010017056.1	*D. avara2*
ENSOUGP00010017062.1	*D. avara2*
ENSOUGP00010017250.1	*D. avara2*
ENSOUGP00010017389.1	*D. avara2*
ENSOUGP00010017540.1	*D. avara2*
ENSOUGP00010017552.1	*D. avara2*
ENSOUGP00010018045.1	*D. avara2*
ENSOUGP00010018051.1	*D. avara2*
ENSOUGP00010018058.1	*D. avara2*
ENSOUGP00010018074.1	*D. avara2*
ENSOUGP00010018127.1	*D. avara2*
ENSOUGP00010018209.1	*D. avara2*
ENSOUGP00010018210.1	*D. avara2*
ENSOUGP00010018217.1	*D. avara2*
ENSOUGP00010018776.1	*D. avara2*
ENSOUGP00010018919.1	*D. avara2*
ENSOUGP00010019117.1	*D. avara2*
ENSOUGP00010019125.1	*D. avara2*
ENSOUGP00010019236.1	*D. avara2*
ENSOUGP00010019250.1	*D. avara2*
ENSOUGP00010020140.1	*D. avara2*
ENSOUGP00010020383.1	*D. avara2*
ENSOUGP00010021435.1	*D. avara2*
ENSOUGP00010022146.1	*D. avara2*
ENSOUGP00010023402.1	*D. avara2*
ENSOUGP00010023426.1	*D. avara2*
ENSOUGP00010024175.1	*D. avara2*
ENSOUGP00010024181.1	*D. avara2*
ENSOUGP00010024306.1	*D. avara2*
ENSOUGP00010024319.1	*D. avara2*
ENSOUGP00010024325.1	*D. avara2*
ENSOUGP00010024365.1	*D. avara2*
ENSOUGP00010024463.1	*D. avara2*
ENSOUGP00010024812.1	*D. avara2*
ENSOUGP00010024943.1	*D. avara2*
ENSOUGP00010024947.1	*D. avara2*

ENSOUGP00010024948.1	*D. avara2*
ENSOUGP00010025475.1	*D. avara2*
ENSOUGP00010025613.1	*D. avara2*
ENSOUGP00010027984.1	*D. avara2*
ENSOUGP00010027990.1	*D. avara2*
ENSOUGP00010028017.1	*D. avara2*
ENSOUGP00010028048.1	*D. avara2*
ENSOUGP00010028058.1	*D. avara2*
ENSOUGP00010028066.1	*D. avara2*
ENSOUGP00010029519.1	*D. avara2*
ENSOUGP00010029948.1	*D. avara2*
ENSOUGP00010029953.1	*D. avara2*
ENSOUGP00010031258.1	*D. avara2*
ENSOUGP00010031590.1	*D. avara2*
ENSOUGP00010031596.1	*D. avara2*
ENSOUGP00010032338.1	*D. avara2*
ENSOUGP00010032344.1	*D. avara2*
ENSOUGP00010032382.1	*D. avara2*
ENSOUGP00010032389.1	*D. avara2*
ENSOUGP00010032395.1	*D. avara2*
ENSOUGP00010032402.1	*D. avara2*
ENSOUGP00010032485.1	*D. avara2*
ENSOUGP00010032666.1	*D. avara2*
ENSOUGP00010032682.1	*D. avara2*
ENSOUGP00010033376.1	*D. avara2*
ENSOUGP00010033384.1	*D. avara2*
ENSOUGP00010034650.1	*D. avara2*
ENSOUGP00010034657.1	*D. avara2*
ENSOUGP00010034759.1	*D. avara2*
ENSOUGP00010034763.1	*D. avara2*
ENSOUGP00010035247.1	*D. avara2*
ENSOUGP00010035261.1	*D. avara2*
ENSOUGP00010035663.1	*D. avara2*
ENSOUGP00010036020.1	*D. avara2*
ENSOUGP00010036079.1	*D. avara2*
ENSOUGP00010036084.1	*D. avara2*
ENSOUGP00010036206.1	*D. avara2*
ENSOUGP00010036378.1	*D. avara2*
ENSOUGP00010036393.1	*D. avara2*
ENSOUGP00010036445.1	*D. avara2*
ENSOUGP00010037290.1	*D. avara2*

ENSOUGP00010037605.1	*D. avara2*
ENSOUGP00010038086.1	*D. avara2*
ENSOUGP00010038091.1	*D. avara2*
ENSOUGP00010038213.1	*D. avara2*
ENSOUGP00010038809.1	*D. avara2*
ENSOUGP00010039139.1	*D. avara2*
ENSOUGP00010039140.1	*D. avara2*
ENSOUGP00010039145.1	*D. avara2*
ENSOUGP00010039518.1	*D. avara2*
ENSOUGP00010039528.1	*D. avara2*
ENSOUGP00010039722.1	*D. avara2*
ENSOUGP00010039934.1	*D. avara2*
ENSOUGP00010039935.1	*D. avara2*
ENSOUGP00010040237.1	*D. avara2*
ENSOUGP00010040442.1	*D. avara2*
ENSOUGP00010040445.1	*D. avara2*
ENSOUGP00010040734.1	*D. avara2*
ENSOUGP00010040739.1	*D. avara2*
ENSOUGP00010040745.1	*D. avara2*
ENSOUGP00010040905.1	*D. avara2*
ENSOUGP00010041154.1	*D. avara2*
ENSOUGP00010041162.1	*D. avara2*
ENSOUGP00010041344.1	*D. avara2*
ENSOUGP00010041715.1	*D. avara2*
ENSOUGP00010042040.1	*D. avara2*
ENSOUGP00010042048.1	*D. avara2*
ENSOUGP00010042051.1	*D. avara2*
ENSOUGP00010042060.1	*D. avara2*
ENSOUGP00010042069.1	*D. avara2*
ENSOUGP00010043289.1	*D. avara2*
ENSOUGP00010043505.1	*D. avara2*
ENSOUGP00010043533.1	*D. avara2*
ENSOUGP00010043565.1	*D. avara2*
ENSOUGP00010043572.1	*D. avara2*
ENSOUGP00010043583.1	*D. avara2*
ENSOUGP00010045177.1	*D. avara2*
ENSOUGP00010045487.1	*D. avara2*
ENSOUGP00010045820.1	*D. avara2*
ENSOUGP00010045831.1	*D. avara2*
ENSOUGP00010046075.1	*D. avara2*

ENSOUGP00010046086.1	*D. avara2*
ENSOUGP00010047311.1	*D. avara2*
ENSOUGP00010047361.1	*D. avara2*
ENSOUGP00010047707.1	*D. avara2*
ENSOUGP00010047712.1	*D. avara2*
ENSOUGP00010047718.1	*D. avara2*
ENSOUGP00010047986.1	*D. avara2*
ENSOUGP00010047995.1	*D. avara2*
ENSOUGP00010048327.1	*D. avara2*
ENSOUGP00010048343.1	*D. avara2*
ENSOUGP00010048449.1	*D. avara2*
ENSOUGP00010048451.1	*D. avara2*
ENSOUGP00010048458.1	*D. avara2*
ENSOUGP00010048467.1	*D. avara2*
ENSOUGP00010048514.1	*D. avara2*
ENSOUGP00010048522.1	*D. avara2*
ENSOUGP00010049242.1	*D. avara2*
ENSOUGP00010049614.1	*D. avara2*
ENSOUGP00010049626.1	*D. avara2*
ENSOUGP00010049630.1	*D. avara2*
ENSOUGP00010049687.1	*D. avara2*
ENSOUGP00010050097.1	*D. avara2*
ENSOUGP00010050191.1	*D. avara2*
ENSOUGP00010051009.1	*D. avara2*
ENSOUGP00010051016.1	*D. avara2*
ENSOUGP00010051204.1	*D. avara2*
ENSOUGP00010051217.1	*D. avara2*
ENSOUGP00010051322.1	*D. avara2*
ENSOUGP00010051527.1	*D. avara2*
ENSOUGP00010052273.1	*D. avara2*
ENSOUGP00010052279.1	*D. avara2*
ENSOUGP00010052301.1	*D. avara2*
ENSOUGP00010052310.1	*D. avara2*
ENSOUGP00010052311.1	*D. avara2*
ENSOUGP00010052319.1	*D. avara2*
ENSOUGP00010052684.1	*D. avara2*
ENSOUGP00010053263.1	*D. avara2*
ENSOUGP00010053383.1	*D. avara2*
ENSOUGP00010053490.1	*D. avara2*
ENSOUGP00010053571.1	*D. avara2*
ENSOUGP00010053815.1	*D. avara2*

ENSOUGP00010053842.1	*D. avara2*
ENSOUGP00010053858.1	*D. avara2*
ENSOUGP00010053862.1	*D. avara2*
ENSOUGP00010053874.1	*D. avara2*
ENSOUGP00010053948.1	*D. avara2*
ENSOUGP00010053952.1	*D. avara2*
ENSRSSP00000006325.1	*C. reniformis*
c1.1824	*D. avara*
c1.2148	*D. avara*
c1.2403	*D. avara*
c1.2404	*D. avara*
c1.81	*D. avara*
c10.555	*D. avara*
c10.648	*D. avara*
c10.708	*D. avara*
c11.1346	*D. avara*
c11.1393	*D. avara*
c11.1422	*D. avara*
c11.1462	*D. avara*
c11.1467	*D. avara*
c11.1475	*D. avara*
c11.407	*D. avara*
c11.668	*D. avara*
c12.1035	*D. avara*
c12.1128	*D. avara*
c12.1514	*D. avara*
c12.1550	*D. avara*
c12.1555	*D. avara*
c12_.225	*D. avara*
c12.278	*D. avara*
c13.102	*D. avara*
c13.1659	*D. avara*
c13.30	*D. avara*
c13.36	*D. avara*
c13.417	*D. avara*
c13.44	*D. avara*
c13.486	*D. avara*
c13.595	*D. avara*
c13.67	*D. avara*
c13.769	*D. avara*

c13.9	*D. avara*
c13.987	*D. avara*
c14.1151	*D. avara*
c14.400	*D. avara*
c14.427	*D. avara*
c14.479	*D. avara*
c14.529	*D. avara*
c15.1096	*D. avara*
c15.1203	*D. avara*
c15.1385	*D. avara*
c15.1394	*D. avara*
c15.47	*D. avara*
c15.472	*D. avara*
c15.780	*D. avara*
c15.93	*D. avara*
c2.10	*D. avara*
c2.1050	*D. avara*
c2.1051	*D. avara*
c2.14	*D. avara*
c2.1908	*D. avara*
c2.205	*D. avara*
c2.48	*D. avara*
c2.51	*D. avara*
c2.56	*D. avara*
c2.711	*D. avara*
c3.1149	*D. avara*
c3.1164	*D. avara*
c3.1200	*D. avara*
c3.1610	*D. avara*
c3.1773	*D. avara*
c3.966	*D. avara*
c4.334	*D. avara*
c4.361	*D. avara*
c5.1101	*D. avara*
c5.1282	*D. avara*
c6.109	*D. avara*
c6.1244	*D. avara*
c6.126	*D. avara*
c6.1323	*D. avara*
c6.1338	*D. avara*
c6.1389	*D. avara*

c6.151	*D. avara*
c6.157	*D. avara*
c6.1648	*D. avara*
c6.1931	*D. avara*
c6.294	*D. avara*
c6.417	*D. avara*
c6.432	*D. avara*
c6.438	*D. avara*
c6.733	*D. avara*
c6.739	*D. avara*
c6.77	*D. avara*
c6.878	*D. avara*
c7.1153	*D. avara*
c7.266	*D. avara*
c7.273	*D. avara*
c7.279	*D. avara*
c7.283	*D. avara*
c7.296	*D. avara*
c7.299	*D. avara*
c7.337	*D. avara*
c7.350	*D. avara*
c7.366	*D. avara*
c7368	*D. avara*
c7.379	*D. avara*
c7.457	*D. avara*
c7.712	*D. avara*
c7.740	*D. avara*
c8.1191	*D. avara*
c8.1237	*D. avara*
c8.128	*D. avara*
c8.142	*D. avara*
c8.170	*D. avara*
c8.1789	*D. avara*
c8.1791	*D. avara*
c8.1819	*D. avara*
c8.1820	*D. avara*
c8.187	*D. avara*
c8.2188	*D. avara*
c8.26	*D. avara*
c8.32	*D. avara*

c8.37	*D. avara*
c8.41	*D. avara*
c8.61	*D. avara*
c8.64	*D. avara*
c8.69	*D. avara*
c8.705	*D. avara*
c9.417	*D. avara*
Nve_NLR_C_XP_048575673.1	*N. vectensis*
Nve_NLR_XP_048578881.1	*N. vectensis*
Nve_NLR_XP_048579698.1	*N. vectensis*
XP_011404815.1	*A. queenslandica*
XP_019852721.1	*A. queenslandica*
XP_019856352.1	*A. queenslandica*
XP_019856353.1	*A. queenslandica*
XP_019856357.1	*A. queenslandica*
XP_019856730.1	*A. queenslandica*
XP_019856732.1	*A. queenslandica*
XP_019856734.1	*A. queenslandica*

Supplementary Table 3. Statistics of the chromosome-level genome assembly of *Dysidea avara.*

D. avara **genome assembly**	**Statistics**
**Total length**	575 Mb
**Number of scaffolds**	162
**Number of chromosomes**	15
**Largest chromosome**	68 Mb
**Number of Ns**	31.3 kb
**GC content**	38%
**N50**	41.2 Mb
**Busco Results for Eukaryota**	C:69.8%[S:68.6%, D:1.2%], F:7.8%, M:22.4%, n:255
**Busco Results for Metazoa**	C:64.9%[S:62.6%, D:2.3%], F:8.8%, M:26.3%, n:954
**Busco Results for Metazoa ASG genome assembly**	C:74.6%[S:72.6%,D:2.0%],F:10.7%,M:14.7%,n:954

**Busco Results for Metazoa annotated genome**	C:77.6%[S:75.3%,D:2.3%],F:3.5%,M:18.9%,n:954
**Busco Results for Metazoa ASG annotated genome**	C:89.1%[S:44.8%,D:44.3%],F:4.1%,M:6.8%,n:954

ASG: Aquatic Symbiosis Genome project

Supplementary Table 4: Blast hits of non-chromosomal sequences in the *D. avara* assembly.

Scaffold_100__1_contigs__length_24027	CP073241.1	Acinetobacter soli strain M3-1-68 chromosome, complete genome
Scaffold_101__1_contigs__length_23867	CP036275.1	Maioricimonas rarisocia strain Mal4 chromosome, complete genome
Scaffold_102__1_contigs__length_23799	CP103298.1	Endozoicomonas gorgoniicola strain PS125 chromosome, complete genome
Scaffold_103__1_contigs__length_23585	CP037918.1	Chromatiaceae bacterium isolate CTD079 chromosome, complete genome
Scaffold_104__1_contigs__length_23372	CP103300.1	Endozoicomonas euniceicola strain EF212 chromosome, complete genome
Scaffold_105__1_contigs__length_23258	CP082909.1	Endozoicomonas sp. 4G chromosome, complete genome
Scaffold_106__1_contigs__length_23220	BX294142.1	Rhodopirellula baltica SH 1 complete genome; segment 10/24
Scaffold_107__1_contigs__length_22456	CP012850.1	Candidatus Nitrosocosmicus oleophilus strain MY3 chromosome, complete genome
Scaffold_108__1_contigs__length_22227	CP036298.1	Aureliella helgolandensis strain Q31a chromosome, complete genome
Scaffold_109__1_contigs__length_22211	CP009533.1	Pseudomonas rhizosphaerae strain DSM 16299, complete genome
Scaffold_110__1_contigs__length_21850	CP076443.1	Gemella sp. zg-570 chromosome, complete genome
Scaffold_111__1_contigs__length_21563	CP002403.1	Ruminococcus albus 7, complete genome
Scaffold_112__1_contigs__length_20961	LK064876.1	Apteryx australis mantelli genome assembly AptMant0, scaffold scaffold150
Scaffold_113__1_contigs__length_20939	FO203363.1	Marinobacter hydrocarbonoclasticus str. ATCC 49840 chromosome, complete genome
Scaffold_114__1_contigs__length_20893	CP019391.1	Brucella sp. 09RB8910 chromosome 2, complete sequence
Scaffold_115__1_contigs__length_20830	CP051300.1	Treponema sp. OMZ 305 chromosome, complete genome

Scaffold_116__1_contigs__length_20321	LC629496.1	MAG: Uncultured marine virus CM1_5m.V42 DNA, partial genome sequence
Scaffold_117__1_contigs__length_20263	KU595465.1	Uncultured virus SERC_370450 chaperonin GroEL (GroEL) gene, complete cds
Scaffold_118__1_contigs__length_20014	CP097384.1	Blastopirellula sp. J2-11 chromosome
Scaffold_119__1_contigs__length_19916	KU756932.1	Uncultured virus SDO_Contig_24668 hypothetical protein, chaperonin GroEL (GroEL), cochaperonin GroES (GroES), and hypothetical protein genes, complete cds
Scaffold_120__1_contigs__length_19859	CP070838.1	MAG: Thiotrichales bacterium isolate bin102 chromosome
Scaffold_121__1_contigs__length_19728	CP047491.1	Microbulbifer hydrolyticus strain IRE-31 chromosome, complete genome
Scaffold_124__1_contigs__length_19294	CP103298.1	Endozoicomonas gorgoniicola strain PS125 chromosome, complete genome
Scaffold_126__1_contigs__length_18754	CP082909.1	Endozoicomonas sp. 4G chromosome, complete genome
Scaffold_128__1_contigs__length_18662	OX244033.2	Tridacna gigas genome assembly, chromosome: 6
Scaffold_131__1_contigs__length_17486	CP076114.1	Pseudomonas seleniipraecipitans strain D1-6 chromosome, complete genome
Scaffold_132__1_contigs__length_17379	CP042914.1	Roseimaritima ulvae strain UC8 chromosome, complete genome
Scaffold_136__1_contigs__length_17168	MK892673.1	MAG: Prokaryotic dsDNA virus sp. isolate Unbinned_5089_contig-100_0, partial genome
Scaffold_138__1_contigs__length_16616	CP058977.1	Streptomyces sp. NEAU-sy36 chromosome, complete genome
Scaffold_139__1_contigs__length_16595	CP013251.1	Endozoicomonas montiporae CL-33 genome
Scaffold_140__1_contigs__length_16578	CP103298.1	Endozoicomonas gorgoniicola strain PS125 chromosome, complete genome
Scaffold_142__1_contigs__length_16465	CP103300.1	Endozoicomonas euniceicola strain EF212 chromosome, complete genome
Scaffold_80__1_contigs__length_28611	MW030542.1	MAG: Virus NIOZ-UU157 genomic sequence, sequence
Scaffold_81__1_contigs__length_28561	MK892533.1	MAG: Prokaryotic dsDNA virus sp. isolate Tp1_58_DCM_122876_1, partial genome
Scaffold_82__1_contigs__length_28452	CP082909.1	Endozoicomonas sp. 4G chromosome, complete genome
Scaffold_83__1_contigs__length_28204	MK892775.1	MAG: Prokaryotic dsDNA virus sp. isolate Tp1_58_DCM_122957_1, complete genome
Scaffold_85__1_contigs__length_27274	CP081025.1	Aeromonas enteropelogenes strain Colony21 chromosome

Supplementary Table 5: Automated annotation of the genome *Dysidea avara* with eggNOG-mapper, separate file.

Supplementary Table 6: Abundance of Pfam domains related to immunity in the chromosome level genomes of 11 sponge species. The number of proteins assigned to a specific domain was taken after independent hmmerscan for each domain based on default parameters and validation with SMART. TIR, Toll/interleukine-2 domain; I-set, Immunoglobilin I-set; V-set, Immunoglobilin V-set; C1-set, Immunoglobilin C1-set, C2-set, Immunoglobilin C2-set; LRR1, Leucine-rich repeat 1 domain; LRR2, Leucin-rich repeat 2 domain; LRR3, Leucin-rich repeat 3 domain; LRRNT, Leucin-rich repeat N-terminal domain; LRCNT, Leucin-rich repeat C-terminal domain; SRCR, Scavenger Receptor cystein rich domain; CARD, Caspase recrutiment domain; DEAD/DEAH box helicase; DED, Death effector domain; LCTD, Lectin C type domain; Fibrin C, Fibrinogen beta and Gamma chains C-terminal globular domain; BIR, Inhibitor of Apoptosis domain; TM, 7 transmembrane (TM) helices_1,2,3.

PFAM-ID	Domain	*A. oroides*	*A. queenslandica*	*A. aerophoba*	*A. vastus*	*C. reniformis*	*C. crambe*	*D. avara*	*Dysida avara2 (ASG)*	*E. muelleri*	*H. panicea*	*O. lobularis*
PF00001	7tm_1	28	88	18	4	13	58	8	132	74	24	62
PF00002	7tm_2	133	84	173	94	86	66	56	400	89	100	42
PF00003	7tm_3	155	61	82	31	54	69	25	111	134	93	26
PF00059	Lectin_C	7	2	2	77	3	3	3	9	1	5	81
PF00147	Fibrin C	52	125	88	0	116	8	59	212	61	22	78
PF00270	DEAD	195	144	183	97	155	111	111	276	189	18	159
PF00530	SRCR	297	363	220	38	191	134	108	214	233	250	131
PF00560	LRR_1	305	39	119	82	126	100	250	562	145	16	172
PF00619	CARD	58	11	63	4	35	20	39	177	29	32	130
PF00653	BIR	5	9	7	5	4	2	15	13	6	6	16
PF01335	DED	11	6	5	2	3	18	7	24	8	18	10
PF01462	LRRNT	5	3	4	1	6	2	1	6	1	4	23
PF01463	LRCNT	0	0	0	0	0	0	0	0	0	0	0
PF01582	TIR	21	3	13	4	11	8	14	33	13	40	33
PF13676	TIR_2	22	3	13	4	11	11	18	32	19	40	40
PF05790	C2-set	25	3	54	7	14	13	209	213	26	12	36
PF07654	C1-set	124	23	68	19	40	30	162	303	24	45	56
PF05729	NACHT	345	70	308	161	217	128	230	369	216	181	144
PF07679	I-set	635	236	511	149	406	205	637	92	315	421	471
PF07686	V-set	434	69	339	66	300	155	564	303	238	283	370
PF07723	LRR_2	49	1	1	4	7	8	9	18	5	13	1
PF07725	LRR_3	2	0	0	1	0	0	6	7	0	2	1
PF08357	SEFIR	4	1	3	2	1	3	2	29	4	4	9

Supplementary Table 7: Domain architecture of the different NLR categories identified in automated annotation of the *D.avara* genome.

NLR category	ID gene	SMART
NLRC	c11.407	(CARD)-NACHT-LRR
NLRC	c14.400	(CARD)-NACHT-LRRx2
NLRC	c6.1648	(CARD)-NACHT-LRRx5
NLRC	c6.1931	(CARD)-NACHT-LRRx6
NLRC	c11.1346	(CARD)-NACHT-LRRX7
NLRC	c15.1203	(CARD-NACHT-LRRx5
NLRD	c7.283	(DEATH)-NACHT-(DUF2075)-LRRx4

NLRD	c6.157	(DEATH)-NACHT-LRRx11
NLRD	c10.648	(DEATH)-NACHT-LRRx22
NLRD	c10.555	(DEATH)-NACHT-LRRx3
NLRD	c2.10	(DEATH)-NACHT-LRRx3
NLRD	c2.205	(DEATH)-NACHT-LRRx3
NLRD	c3.1610	(DEATH)-NACHT-LRRx3
NLRD	c3.1773	(DEATH)-NACHT-LRRx3
NLRD	c8.142	(DEATH)-NaCHT-LRRx3
NLRD	c8.1789	(DEATH)-NACHT-LRRx3
NLRD	c8.2188	(DEATH)-NACHT-LRRx3
NLRD	c8.61	(DEATH)-NaCHT-LRRx3
NLRD	c8.1191	(DEATH)-NaCHT-LRRx5
NLRD	c9.592	(DEATH)-NACHT-LRRx9
NLRC	c11.1306	CARD-NACHT-(LRR)
NLRC	c11.240	CARD-NACHT-(LRR)
NLRC	c14.399	CARD-NACHT-LRR
NLRC	c15.6	CARD-NACHT-LRR
NLRC	c7.457	CARD-NACHT-LRRx3
NLRC	c1.2404	CARD-NACHT-LRRx4
NLRC	c14.427	CARD-NACHT-LRRx4
NLRC	c7.350	CARD-NACHT-LRRx5
NLRC	c7.368	CARD-NACHT-LRRx5
NLRC	c11.1475	CARD-NACHT-LRRX6
NLRC	c1.2403	CARD-NACHT-LRRx6
NLRC	c6.109	CARD-NACHT-LRRx7
NLRC	c11.1393	CARD-NACHT-LRRX7-tm
NLRC	c14.479	CARD-NACHT-LRRx8
NLRD	c15.47	DEATH-NACHT-LRR
NLRD	c2.14	DEATH-NACHT-LRRx2
NLRD	c15.472	DEATH-NACHT-LRRx5
NLRD	c6.294	DEATH-NACHT-LRRx5
NLRD	c6.77	DEATH-NACHT-LRRx5
NLRD	c7.273	DEATH-NACHT-LRRx5
NLRD	c7.279	DEATH-NACHT-LRRx5
NLRD	c7.299	DEATH-NACHT-LRRx6
NLRD	c8.1820	RPT1(DEATH)-RPT1(DEATH)-NACHT-LRRX3
NLRD	c7.1832	TM-(CARD)-NACHT-LRR-LITAF
NLRD	c15.394	TNFR-TNFR-TNFR-DEATH-NACHT-(LRR)
NLRX	c7.266	UBQ-NACHT-LRRx5
NLRX	c7.296	NACHT-AAA-LRRX4
NLRX	c5.375	NACHT-LRR
NLRX	c13.486	NACHT-LRRx10
NLRX	c6.126	NACHT-LRRx10

NLRX	c6.151	NACHT-LRRx12
NLRX	c6.878	NACHT-LRRx12
NLRX	c2.1051	NACHT-LRRx14
NLRX	c11.668	NACHT-LRRX16
NLRX	c3.1164	NACHT-LRRx17
NLRX	c12.1514	NACHT-LRRx2
NLRX	c13.36	NACHT-LRRx2
NLRX	c14.1151	NACHT-LRRX2
NLRX	c15.93	NACHT-LRRx2
NLRX	c6.1389	NACHT-LRRx2
NLRX	c8.128	NACHT-LRRx2
NLRX	c8.1791	NACHT-LRRx2
NLRX	c8.1819	NACHT-LRRx2
NLRX	c2.1050	NACHT-LRRx21
NLRX	c12.1035	NACHT-LRRX3
NLRX	c12.278	NACHT-LRRX3
NLRX	c1.2148	NACHT-LRRx3
NLRX	c13.30	NACHT-LRRx3
NLRX	c13.595	NACHT-LRRx3
NLRX	c13.987	NACHT-LRRx3
NLRX	c2.1462	NACHT-LRRx3
NLRX	c2.48	NACHT-LRRx3
NLRX	c2.51	NACHT-LRRx3
NLRX	c2.56	NACHT-LRRx3
NLRX	c4.334	NACHT-LRRx3
NLRX	c4.361	NACHT-LRRx3
NLRX	c7.1153	NACHT-LRRx3
NLRX	c8.187	NACHT-LRRx3
NLRX	c8.69	NACHT-LRRx3
NLRX	c8.705	NACHT-LRRx3
NLRX	c8.41	NACHT-LRRx3-(DUF4041)
NLRX	c8.1237	NACHT-LRRx30
NLRX	c2.711	NACHT-LRRx3-ITI_HC_C
NLRX	c8.33	NACHT-LRRx3-NACHT-LRRx4-PHD
NLRX	c13.44	NACHT-LRRx4
NLRX	c2.1908	NACHT-LRRx4
NLRX	c3.1149	NACHT-LRRx4
NLRX	c7.740	NACHT-LRRx4
NLRX	c8.170	NACHT-LRRx4
NLRX	c8.26	NACHT-LRRx4
NLRX	c8.32	NACHT-LRRx4
NLRX	c8.37	NACHT-LRRx4
NLRX	c8.64	NACHT-LRRx4
NLRX	c12.1128	NACHT-LRRx4-RPT1

NLRX	c1.1824	NACHT-LRRx5
NLRX	c13.417	NACHT-LRRx5
NLRX	c3.966	NACHT-LRRx5
NLRX	c6.417	NACHT-LRRx5
NLRX	c6.432	NACHT-LRRx5
NLRX	c7.366	NACHT-LRRx5
NLRX	c7.379	NACHT-LRRx5
NLRX	c15.1385	NACHT-LRRx6
NLRX	c3.1200	NACHT-LRRx6
NLRX	c7.712	NACHT-LRRx6
NLRX	c11.1467	NACHT-LRRX7
NLRX	c12.225	NACHT-LRRX7
NLRX	c13.1659	NACHT-LRRX7
NLRX	c15.1394	NACHT-LRRx7
NLRX	c11.1422	NACHT-LRRX8
NLRX	c11.1462	NACHT-LRRX8
NLRX	c11.885	NACHT-LRRX8
NLRX	c14.529	NACHT-LRRX8
NLRX	c15.1096	NACHT-LRRx8
NLRX	c6.733	NACHT-LRx5
NLRX	c6.739	NACHT-LRx7
NLRX	c12.1550	NACHT-NACHT-LRRx12
NLRX	c15.780	NACHT-RPT1-RPT1-LRRx9
NLRX	c6.1338	NACHT-TM-LRx4
NLRX	c6.438	NACHT-TM-LRx7
NLRX	c1.948	NACHT-TSR(LRR)
NLRX	c6.1323	NACHT-LRRx5-(DEATH) or (intBPP-1)
NLRX	c13.102	SIGNALP-NACHT-LRR3
NLRX	c10.708	(NACHT)-LRRx10
NLRX	c12.1555	KIX_2-NACHT-LRRX24
NLRX	c4.226	NACHT-(Fbox-like)-LRR
NLRX	c5.1282	NACHT-LRR

Supplementary Table 8: Position of the different NLR categories across the 15 chromosomes of *D. avara* genome.

**NLR category**	**ID gene**	**Chr**	**Start**	**End**
NLRC	c11.407	11	8663114	8671483
NLRC	c14.400	14	2719428	2722816
NLRC	c6.1648	6	36999020	37002342
NLRC	c6.1931	6	43564632	43567554
NLRC	c11.1346	11	26787289	26815922
NLRC	c15.1203	15	17725090	17742884
NLRD	c7.283	7	4326607	4358878

NLRD	c6.157	6	2728926	2746515
NLRD	c10.648	10	8954147	8964687
NLRD	c10.555	10	7237704	7240697
NLRD	c2.10	2	99736	102463
NLRD	c2.205	2	3802770	3805605
NLRD	c3.1610	3	35000711	35012600
NLRD	c3.1773	3	38673630	38683515
NLRD	c8.142	8	2011532	2014326
NLRD	c8.1789	8	33206052	33223203
NLRD	c8.2188	8	39194041	39258055
NLRD	c8.61	8	1056744	1059398
NLRD	c8.1191	8	21141249	21151980
NLRD	c9.592	9	13427114	13428655
NLRC	c11.1306	11	26364593	26384475
NLRC	c11.240	11	5198469	5204576
NLRC	c14.399	14	2704028	2713138
NLRC	c15.6	15	11984	18863
NLRC	c7.457	7	7710779	7728128
NLRC	c1.2404	1	60025720	60029186
NLRC	c14.427	14	3007886	3021764
NLRC	c7.350	7	5808112	5822519
NLRC	c7.368	7	6116392	6139723
NLRC	c11.1475	11	28754742	28765231
NLRC	c1.2403	1	59996782	60000093
NLRC	c6.109	6	1900009	1911167
NLRC	c11.1393	11	27480970	27495471
NLRC	c14.479	14	3348686	3353482
NLRD	c15.47	15	450144	454800
NLRD	c2.14	2	131185	133916
NLRD	c15.472	15	6803478	6814612
NLRD	c6.294	6	5263099	5267781
NLRD	c6.77	6	1205319	1253247
NLRD	c7.273	7	4125823	4141474
NLRD	c7.279	7	4246927	4271180
NLRD	c7.299	7	4705603	4739590
NLRD	c8.1820	8	33773911	33786464
NLRD	c7.1832	7	30230577	30261224
NLRD	c15.394	15	5607123	5611462
NLRX	c7.296	7	4581411	4614775
NLRX	c5.375	5	4340497	4342779
NLRX	c13.486	13	6990878	7013802
NLRX	c6.126	6	2212452	2226050
NLRX	c6.151	6	2643583	2646252
NLRX	c6.878	6	19079675	19083277

NLRX	c2.1051	2	23921443	23926153
NLRX	c11.668	11	14826073	14850547
NLRX	c3.1164	3	25768925	25797870
NLRX	c12.1514	12	19656758	19688255
NLRX	c13.36	13	542126	545322
NLRX	c14.1151	14	14980886	14985640
NLRX	c15.93	15	940148	942509
NLRX	c6.1389	6	31887354	31952749
NLRX	c8.128	8	1803456	1806260
NLRX	c8.1791	8	33224460	33226812
NLRX	c8.1819	8	33766310	33773665
NLRX	c2.1050	2	23862864	23905999
NLRX	c12.1035	12	14249696	14277426
NLRX	c12.278	12	4669483	4672878
NLRX	c1.2148	1	54786258	54788390
NLRX	c13.30	13	454313	457531
NLRX	c13.595	13	8975271	8982348
NLRX	c13.987	13	15012002	15015671
NLRX	c2.1462	2	36122572	36124926
NLRX	c2.48	2	974050	976194
NLRX	c2.51	2	1035246	1037390
NLRX	c2.56	2	1124179	1126479
NLRX	c4.334	4	6240227	6254149
NLRX	c4.361	4	6588006	6590171
NLRX	c7.1153	7	20330149	20334429
NLRX	c8.187	8	2537191	2539996
NLRX	c8.69	8	1227165	1229946
NLRX	c8.705	8	11725514	11729171
NLRX	c8.41	8	824264	827076
NLRX	c8.1237	8	22099483	22105224
NLRX	c2.711	2	15580911	15647137
NLRX	c8.33	8	663145	698313
NLRX	c13.44	13	630300	633465
NLRX	c2.1908	2	49044943	49070169
NLRX	c3.1149	3	25386540	25407135
NLRX	c7.740	7	13671356	13684125
NLRX	c8.170	8	2275397	2278239
NLRX	c8.26	8	553945	556752
NLRX	c8.32	8	613852	615963
NLRX	c8.37	8	770403	772514
NLRX	c8.64	8	1122905	1125686
NLRX	c12.1128	12	14964649	14968529
NLRX	c1.1824	1	46272034	46289979
NLRX	c13.417	13	6026064	6029466
NLRX	c3.966	3	21174812	21184090

NLRX	c6.417	6	8344779	8407712
NLRX	c6.432	6	8790630	8837162
NLRX	c7.366	7	6075792	6078680
NLRX	c7.379	7	6229948	6232563
NLRX	c15.1385	15	20804500	20830675
NLRX	c3.1200	3	26712556	26723363
NLRX	c7.712	7	12905709	12934589
NLRX	c11.1467	11	28641614	28655270
NLRX	c12.225	12	3571429	3574782
NLRX	c13.1659	13	26232768	26239237
NLRX	c15.1394	15	20971244	20974240
NLRX	c11.1422	11	27918195	27929815
NLRX	c11.1462	11	28575106	28585455
NLRX	c11.885	11	19691388	19705543
NLRX	c14.529	14	3918602	3921274
NLRX	c15.1096	15	16628251	16634215
NLRX	c6.733	6	15902117	15916794
NLRX	c6.739	6	16001322	16004033
NLRX	c12.1550	12	20261555	20308595
NLRX	c15.780	15	11478517	11508252
NLRX	c6.1338	6	30857587	30865582
NLRX	c6.438	6	8973459	9054265
NLRX	c1.948	1	22534712	22546114
NLRX	c6.1323	6	30548532	30552398
NLRX	c13.102	13	1419403	1421694
NLRX	c10.708	10	10153154	10165208
NLRX	c12.1555	12	20353562	20410788
NLRX	c4.226	4	4024710	4027085
NLRX	c5.1282	5	16881719	16892377

Supplementary Table 9: Domain architecture of NACHT-containing proteins not related to NLRs.

Gene	Domain Architecture
evm.model.c5.561	AAA-NACHT
evm.model.c13.769	AAA-Rdp-NACHT
evm.model.c8.227	ABCmembrane-NACHT-ABCmembrane-NACHT
evm.model.c8.228	ABCmembrane-NACHT-ABCmembrane-NACHT
evm.model.c2.1423	Ankx4-RPT1x4-NACHT-NACHT
evm.model.c3.143	BAH-NACHT-cdc6
evm.model.c2.927	CARD-NACHT-ZU5
evm.model.c2.1753	CARD-RPTx4-NACHT

evm.model.c3.1323	CDC48-CDC48-AAA-NACHT-Vps4
evm.model.c3.61	CUE-NACHT-DUF-SMR
evm.model.c12.1007	DEAD-Helic-HA2-TUDOR
evm.model.c8.1375	NACHT-AAA
evm.model.c5.809	NACHT-AAA-RT2
evm.model.c6.1427	NACHT-AAA-TM
evm.model.c4.33	NACHT-AAA-Vps4
evm.model.c7.1886	NACHT-ABCmembrane
evm.model.c7.2044	NACHT-ABCmembrane
evm.model.c7.2048	NACHT-ABCmembrane
evm.model.c7.2051	NACHT-ABCmembrane
evm.model.c7.1889	NACHT-ABCmembrane-ANFreceptor-7TM
evm.model.c5.1267	NACHT-Bromodomain
evm.model.c5.43	NACHT-HA2-OB_NTP_bind
evm.model.c13.106	NACHT-HTPase-DNAmisrepair-MutLc
evm.model.c12.2119	Nacht-IG-CARD-IG-Death
evm.model.c12.1018	NACHT-NACHT
evm.model.c7.1155	NACHT-NACHT-RPT1
evm.model.c8.709	NACHT-PeptidaseM41
evm.model.c14.553	NACHT-RPT1-RPT2-AAA
evm.model.c15.767	NACHT-SAM
evm.model.c8.1102	NACHT-SAM
evm.model.c8.1388	NACHT-SAM-SAM
evm.model.c13.398	NACHT-TPR
evm.model.c13.396	NACHT-TPRx4
evm.model.c13.1579	NACHT-VWA
evm.model.c6.1204	NACHT-WD40x13
evm.model.c8.1303	NACHT-WD40x9
evm.model.c14.209	PDB4I9B-NACHT-ClpB
evm.model.c15.578	PHD-NACHT-DSRM-CASc
evm.model.c13.12	PHTB1_N-PHTB1_C-NACHT-4k17
evm.model.c7.701	PNPUDP-NACHT
evm.model.c15.1086	PrmA-NACHT
evm.model.c12.620	RAS-NACHT-KU-TIL
evm.model.c15.1189	RLI-Fer4-AAA-NACHT
evm.model.c11.1514	RPT1-ADH-NACHT
evm.model.c12.267	RPT1-RPT1-Death-NACHT

evm.model.c12.267	RPT1-RPT1-Death-NACHT
evm.model.c6.677	RPT1-RPT1-DEATH-NACHT
evm.model.c12.307	RPT1-RPT1-NACHT
evm.model.c8.515	RPT1-RPT1-NACHT
evm.model.c8.1434	RPT1-RPT1-NACHT
evm.model.c2.1369	RPT1-RPT1-NACHT
evm.model.c2.1371	RPT1-RPT1-NACHT
evm.model.c2.1801	RPT1-RPT1-NACHT
evm.model.c2.1921	RPT1-RPT1-NACHT
evm.model.c8.1429	RPT1-RPT1-NACHT-SAM
evm.model.c11.1514	RPT-ADH-NACHT
evm.model.c12.307	RPt-RPT-NACHT
evm.model.c12.323	RPt-RPT-NACHT
evm.model.c1.81	STYK-COR-DEATH-NACHT-LRRx9
evm.model.c13.75	SUI-PHTB1_N-PHTB1_C-NACHT
evm.model.c2.1868	sushi-sushi-sushi-NACHT
evm.model.c12.175	TIG-NACHT
evm.model.c4.30	TM-NACHT-TPR
evm.model.c14.846	Toprim-NACHT
evm.model.c11.92	NACHT
evm.model.c11.955	NACHT
evm.model.c11.1028	NACHT
evm.model.c11.1431	NACHT
evm.model.c11.1559	NACHT
evm.model.c12.223	NACHT
evm.model.c12.242	NACHT
evm.model.c12.490	NACHT
evm.model.c12.490	NACHT
evm.model.c12.1019	NACHT
evm.model.c13.368	NACHT
evm.model.c13.1430	NACHT
evm.model.c14.341	NACHT
evm.model.c14.482	NACHT
evm.model.c14.1398	NACHT
evm.model.c15.439	NACHT
evm.model.c15.1392	NACHT
evm.model.c15.1849	NACHT

evm.model.c15.1879	NACHT
evm.model.c5.400	NACHT
evm.model.c5.567	NACHT
evm.model.c5.721	NACHT
evm.model.c5.726	NACHT
evm.model.c5.805	NACHT
evm.model.c6.673	NACHT
evm.model.c6.1011	NACHT
evm.model.c7.1192	NACHT
evm.model.c8.273	NACHT
evm.model.c8.280	NACHT
evm.model.c8.296	NACHT
evm.model.c8.369	NACHT
evm.model.c8.1071	NACHT
evm.model.c8.1171	NACHT
evm.model.c8.1371	NACHT
evm.model.c8.1399	NACHT
evm.model.c8.1706	NACHT
evm.model.c8.1930	NACHT
evm.model.c10.164	NACHT
evm.model.c10.231	NACHT
evm.model.c10.722	NACHT
evm.model.c10.952	NACHT
evm.model.c10.1043	NACHT
evm.model.c10.1319	NACHT
evm.model.c10.1373	NACHT
evm.model.c1.37	NACHT
evm.model.c1.73	NACHT
evm.model.c1.83	NACHT
evm.model.c1.1247	NACHT
evm.model.c1.2040	NACHT
evm.model.c2.838	NACHT
evm.model.c2.972	NACHT
evm.model.c2.1340	NACHT
evm.model.c2.1365	NACHT
evm.model.c2.1366	NACHT
evm.model.c2.1367	NACHT

evm.model.c2.1378	NACHT
evm.model.c2.1666	NACHT
evm.model.c2.1800	NACHT
evm.model.c2.1847	NACHT
evm.model.c2.1859	NACHT
evm.model.c2.1917	NACHT
evm.model.c3.5	NACHT
evm.model.c3.157	NACHT
evm.model.c3.689	NACHT

Supplementary Figure 1: Heat map of Omni-C vs. HiRise alignment depicting the 15 largest chromosomes of the genome assembly of *Dysidea avara*.

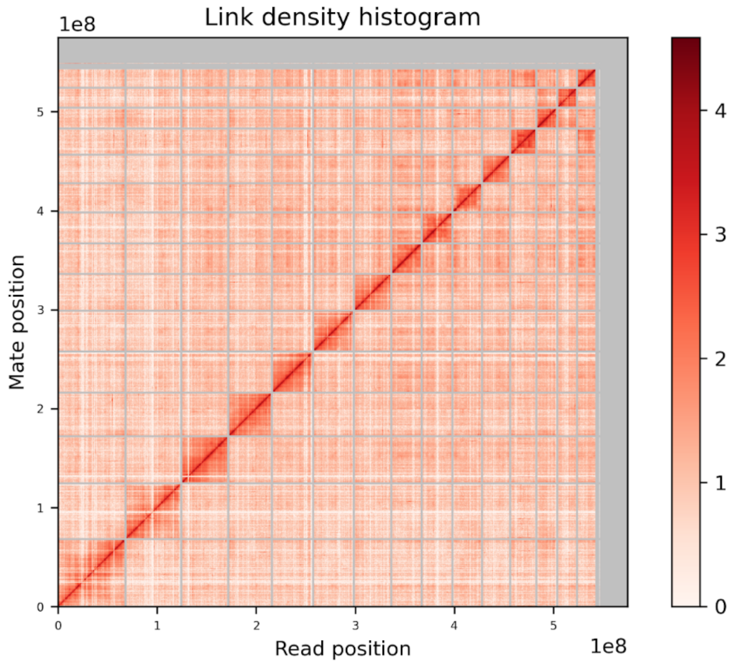

Supplementary Figure 2: Genome size calculation of the genome *Dysidea avara* with GenomeScope.

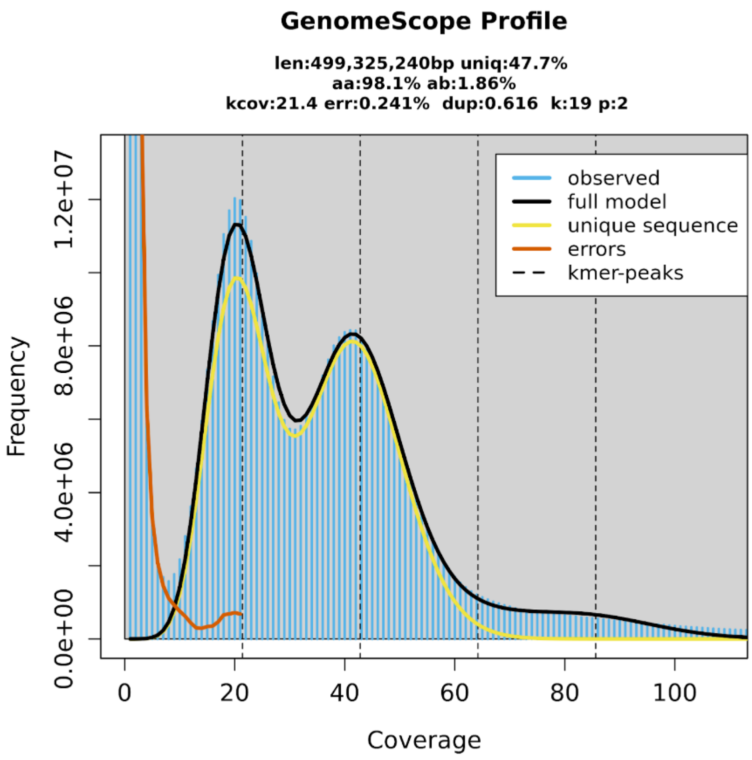

Supplementary Figure 3. NLR sponge repertoires. A) genome assembly spans and total of NACHT-containing proteins. **B)** Total bona fide NLR repertoire and counts for each category (NLRX, NLRD, NLRC). NLRC (CARD–NACHT-LRR), NLRD (DEATH-NACHT-LRRs) and NLRX (NACHT- LRR) *D. avara*: *Dysidea avara*; *D.avara* v2-ASG: *Dysidea avara* genome from Sanger*; C. crambe*: *Crambe crambe*; *C. reniformis*: *Chondrosia reniformis*; *A. oroides*: *Agelas oroides*; *A. aerophoba*: *Aplysina aerophoba*; *A. queensalndica*: Amphimedon queenslandica; H. panicea: *Halichondria panicea*; *E. muelleri*: *Ephydatia muelleri*; *O. lobularis*: *Oscarella lobularis*; *A. vastus*: *Aphrocallistes vastus.*

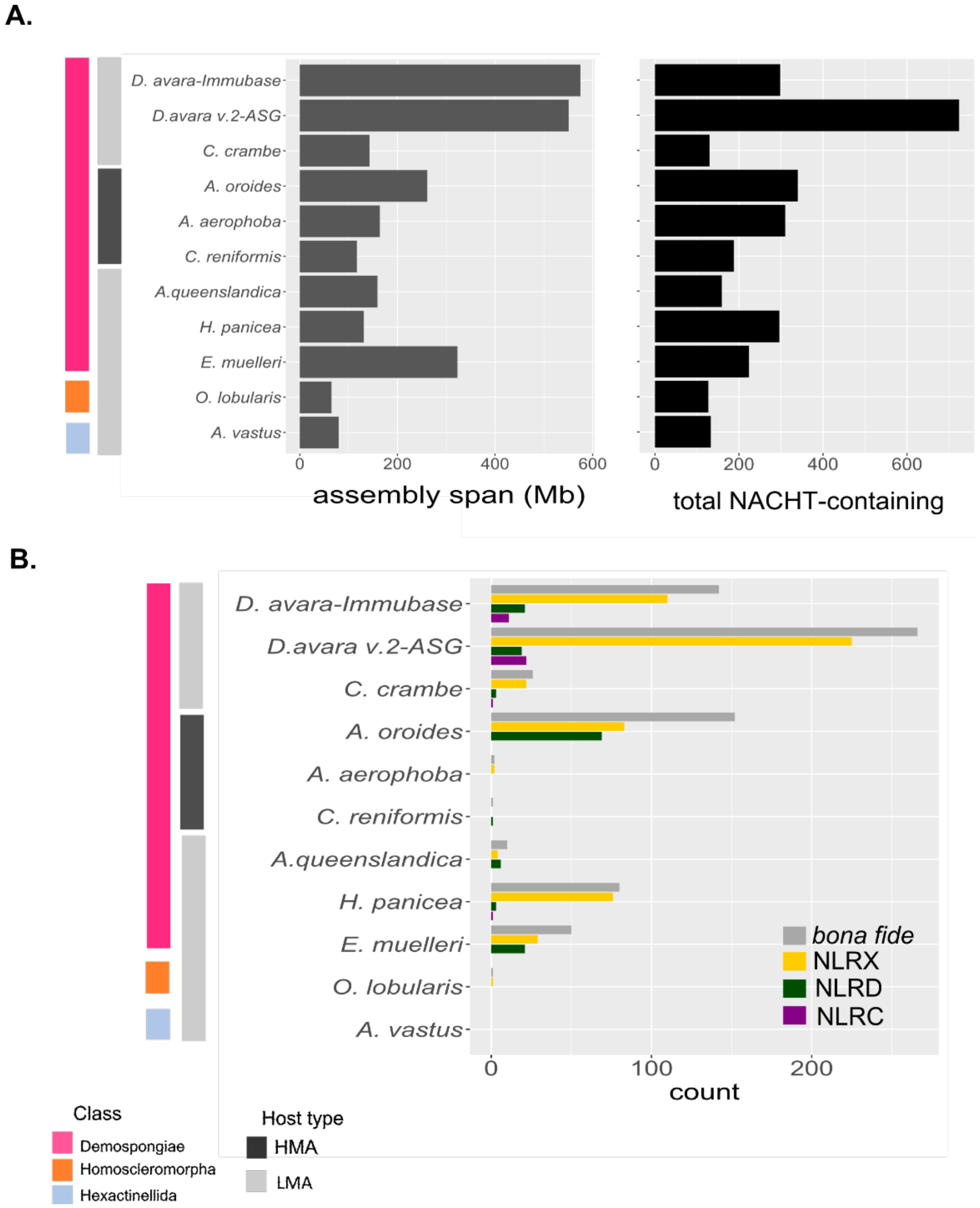

REFERENCES

1. Srivastava M, Simakov O, Chapman J, Fahey B, Gauthier MEA, Mitros T, et al. The *Amphimedon queenslandica* genome and the evolution of animal complexity. Nature [Internet]. 2010;466(7307):720–6. Available from: http://dx.doi.org/10.1038/nature09201

2. Pita L, Hentschel U, Steindler L, Maldonado M, Riesgo A, Oatley G, et al. The chromosomal genome sequence of *Aplysina aerophoba* (Nardo, 1833) and its associated microbial metagenome sequences. Wellcome Open Res. 2025 May 19;10:250.

3. Francis WR, Eitel M, Vargas S, Garcia-Escudero CA, Conci N, Deister F, et al. The genome of the reef-building glass sponge *Aphrocallistes vastus* provides insights into silica biomineralization. R Soc Open Sci. 2023 Jun 21;10(6).

4. Pita L, Maldonado M, Koutsouveli V, Riesgo A, Hentschel U, Oatley G, et al. The chromosomal genome sequence of the kidney sponge, *Chondrosia reniformis* Nardo, 1847, and its associated microbial metagenome sequences. Wellcome Open Res. 2025 May 29;10:283.

5. Maldonado M, Pita L, Hentschel U, Erpenbeck D, Oatley G, Sinclair E, et al. The chromosomal genome sequence of the sponge *Crambe crambe* (Schmidt, 1862) and its associated microbial metagenome sequences. Wellcome Open Res. 2025 May 23;10:275.

6. Kenny NJ, Francis WR, Rivera-Vicéns RE, Juravel K, de Mendoza A, Díez-Vives C, et al. Tracing animal genomic evolution with the chromosomal-level assembly of the freshwater sponge *Ephydatia muelleri*. Nat Commun. 2020 Jul 27;11(1):3676.

7. Riesgo A, Renard E, Schenkelaars Q, Borchiellini C, Pita L, Maldonado M, et al. The chromosomal genome sequence of the sponge, *Oscarella lobularis* (Schmidt, 1862) (Porifera, Homoscleromorpha) and its associated microbial metagenome sequences. Wellcome Open Res. 2025 Jul 9;10:340.
